# Family-authored ICU diaries to reduce fear in patients experiencing a cardiac arrest (FAID fear): A pilot randomized controlled trial

**DOI:** 10.1371/journal.pone.0288436

**Published:** 2023-07-27

**Authors:** Talea Cornelius, Miguel Mendieta, Robin M. Cumella, David Lopez Veneros, Isabella M. Tincher, Sachin Agarwal, Ian Kronish

**Affiliations:** 1 Center for Behavioral Cardiovascular Health, Columbia University Irving Medical Center, New York, New York, United States of America; 2 School of Nursing, Columbia University Irving Medical Center, New York, New York, United States of America; 3 Department of Neurology, Columbia University Irving Medical Center, New York, New York, United States of America; CHU Nantes, FRANCE

## Abstract

Survivors of cardiac arrest (CA) and their family members often experience significant fear-based distress (cardiac fear; i.e., fear about the CA survivor’s heart). Fear-based distress after CA is associated with higher rates of cardiac event recurrence and mortality in CA survivors. As posited in Dyadic Disruption Theory (DDT), cardiac fear in family members may contribute to the development of distress in CA survivors via socially-based mechanisms. Thus, interventions to reduce family distress may improve CA survivors’ outcomes. ICU diaries are easy to implement and scalable and show promise for reducing distress after CA but are primarily targeted towards survivors. The primary aim of the Family-Authored ICU Diaries to reduce Fear in Patients Experiencing a CA (FAID Fear) pilot randomized controlled trial was to test feasibility of an ICU diary intervention targeted towards family member distress alone. Family members of patients hospitalized after CA (*N* = 16) were randomized 2:1 to receive the FAID Fear intervention or usual care. Intervention participants were provided brief instructions and were asked to write in the diary twice per week until the end of hospital care. Assessments occurred at baseline enrollment, end of hospital care, and 30 days later. Participants’ mean age was 50.73 years (*SD* = 13.41; 80% cis-gender female; 60% White). Recruitment (16/25 referred; 64.0%), retention (14/16 enrolled; 87.5%), and intervention adherence (7/10 completed; 70%) were promising. Most agreed that the ICU diary intervention was appropriate (7/10 completed; 70.0%), feasible (9/10 completed; 90.0%]), and acceptable (8/10 completed; 80.0%). Fear was nonsignificantly lower in intervention participants (v. control) at end of hospital care and 30 days later. FAID Fear represents a first step in building theory-based dyadic interventions that can be implemented to support family members of CA survivors in the ICU, with potential to improve outcomes in CA survivors.

## Introduction

Psychological distress is common in patients who survive a cardiac arrest (CA) and their family members [1–7]. In fact, meta-analytic evidence suggests that clinically significant posttraumatic stress symptoms (PTSS) secondary to a CA may be even more common in family members than in patients themselves [4, 7–9]. Critically, CA survivors who develop PTSS after CA experience higher rates of cardiac event recurrence and mortality [1, 2], likely due in part to avoidance of health behaviors for secondary prevention (e.g., fear of physical activity, greater sedentary behavior) [10–14]. Survivors’ distress early after CA is a known contributor to the development of PTSS [12, 13].

Whereas associations of CA survivors’ distress, and PTSS in particular, with health and well-being is more established, the contribution of family members’ distress to CA survivors’ outcomes is not well understood. Dyadic Disruption Theory (DDT) [15] describes the ways in which distressed family members may unintentionally exacerbate distress in CA survivors via mechanisms of emotion contagion [16, 17], unskilled support attempts [18, 19], and co-construction of memories [20–22] as survivors work to make sense of the CA and associated stay in the hospital and intensive care unit (ICU). Supportive evidence from other cardiac patient populations shows that those individuals who arrive in the emergency department with their family members report receiving unhelpful forms of social support, which contributes to early distress and PTSS one month later [18, 23]. Thus, reducing family members’ distress is an unexplored yet potentially powerful socially-based mechanism for reducing survivor distress after CA and, consequently, improving patient outcomes.

ICU diaries comprise notes written by nursing staff, other medical personnel, family, and/or friends, and are intended to help survivors understand their illness, increase involvement in treatment, and improve mental health and well-being [9]. ICU diaries may reduce distress, including PTSS, in family members as well [24–26], but previous ICU diary interventions have been implemented to give the diary to survivors, with no attention to socially-based mechanisms of distress transmission. Given the ease of implementation, potential for scalability, and lack of evidence-based interventions to support family members of ICU survivors, family-authored ICU diaries are a promising intervention for reducing distress in family members. Yet, before conducting a full trial, there are important feasibility questions. It is unknown whether family members, who often initially focus solely on the needs of the CA survivor [27, 28], will find an intervention intended to reduce their own distress feasible, acceptable, and appropriate. Even if family members consent to such a study, it is unclear whether they will engage with the diary.

This pilot randomized controlled trial (RCT) tested the feasibility of the Family-Authored ICU Diaries to reduce Fear in Patients Experiencing a Cardiac Arrest (FAID Fear; NCT05144477) intervention. Primary aims were to estimate recruitment and retention rates, and secondary aims were to estimate intervention compliance, adherence to study procedures, and participant-reported feasibility, acceptability, and appropriateness of the intervention. Results of this pilot RCT will inform intervention refinement and the feasibility of conducting a larger RCT that assesses the efficacy of FAID Fear for reducing family members’ distress, including ongoing cardiac fear about the CA survivor’s heart (e.g., worry even when tests are normal, fear or worry when experiencing a rapid heartbeat) and PTSS related to the CA, with the ultimate goal of improving CA survivors’ mental and physical health outcomes. The novel focus of FAID Fear will contribute to the understanding of the feasibility of interventions targeting socially-based mechanisms that may undermine patient well-being after CA and ICU stay.

## Materials and methods

### Design

This study was a two-arm parallel group pilot RCT comparing a family-authored ICU diary intervention to a usual-care control group. Unequal randomization of 2:1 was selected to maximize data regarding the feasibility of the ICU diary intervention while still testing the feasibility of randomization.

### Setting

This study was conducted at Columbia University Irving Medical Center (CUIMC), a high-volume, tertiary-care referral facility located in New York City that follows the guideline-concordant post-CA care [29] for all patients. Recruitment took place from November 22, 2021—September 30, 2022.

### Eligibility criteria

Participants were recruited from the Cardiac Neuropsychosocial Outcomes Evaluation–Family (CANOE-F; R01 HL153311; PI Agarwal) observational cohort study of family members of CA patients within CUIMC/NewYork-Presbyterian Hospital (NYPH). Family members who self-identified as the primary caregiver for the patient were approached 48 hours or longer after their loved one’s CA, while the patient was in the ICU or after inpatient transfer, and offered participation in CANOE-F.

CANOE-F participants were offered enrollment into FAID Fear if they (1) agreed to be contacted about future research opportunities and (2) enrolled into CANOE-F while the patient was still in the ICU, given that this diary intervention was designed to be implemented during ICU stay. This recruitment strategy was implemented to build trust and remain sensitive to the wishes of family members regarding research participation during this stressful time. FAID Fear eligibility criteria additionally included (1) primary partner or family member of a patient who was hospitalized at CUIMC for a CA, (2) age 18 years or over, (3) able to speak, read, and write in English or Spanish, (4) willing to write in a journal about their experiences, and (5) available for follow-up. Participants were excluded if they presented any medical or psychiatric impairment that would have prevented them from complying with the research protocol.

### Recruitment and consent

CANOE-F participants who met FAID Fear eligibility criteria were referred to study staff and approached either in person or by telephone, depending upon preference and availability. Bilingual study staff then reviewed a verbal information sheet that provided an overview of what was involved in study participation in either English or Spanish, and a copy of the information sheet was given to each participant. All study procedures were approved by the CUIMC Institutional Review Board, and the study was registered on clinicaltrials.gov prior to recruitment (NCT05144477). All participants provided verbal informed consent.

### Randomization and allocation

Participants were randomly allocated in a 2:1 ratio to either the diary intervention or to the control group. This was accomplished by shuffling 16 opaque, sealed envelopes, 11 of which allocated participants to the diary intervention and 5 of which allocated participants to the control group. Allocation remained fully concealed until participants provided verbal informed consent, after which study staff opened the envelopes containing the randomization assignment and informed the participant of their allocation. After this point, due to study design and resource limitations, family members, outcome assessors, and data analysts were aware of allocation. The same study staff were responsible for randomization generation, allocation, and enrollment.

### Study visits

Participants in both the diary intervention and the control group completed three study visits: (1) a baseline/pre-intervention session, (2) a post-intervention session at the end hospital care for the CA, and (3) a follow-up session 30 days after the end of hospital care. During the baseline/pre-intervention session, participants provided verbal consent, were randomized, and completed a questionnaire. Participants randomized to the intervention group were given verbal and written instructions to complete the ICU diary, received a hard-cover diary and pen, and were asked to start writing in the ICU diary.

The post-intervention session was conducted over the phone and occurred only if the CA patient survived and was discharged from the hospital. During this session, participants completed study questionnaires. The intervention ended at this time, and intervention participants were informed that continued usage of the ICU diary was optional.

The follow-up session was also conducted over the phone. At this final assessment, participants again completed study questionnaires. Participants were compensated up to $100 for their time. Participants received $50 after completing the baseline/pre-intervention session ($25 for randomization and $25 for completing the baseline survey) and $50 for survey completion at the follow-up session. Payment was not contingent on adherence to the diary intervention.

### Intervention

Following the design of Nielsen et al. [30], participants randomized to the ICU diary intervention were provided a hard-cover diary, pen, and ICU diary instructions (see S1 Appendix). Instructions included recommended frequency for writing, guidance on potential topics, and recommendations for sharing the diary with the patient (if desired). Participants were asked to continue writing in the diary, preferably twice a week, until the end of hospital care, with no suggested length per entry. Nielsen et al. [30] instructed participants to write “often” and at any length; in this study, twice a week was suggested based on feedback from key stakeholders (CA survivors, family members of CA survivors) to provide more concrete guidelines while not suggesting a frequency that was potentially burdensome. Research staff conducted weekly check-ins by phone with participants throughout patient hospitalization to answer any questions regarding usage of the diary and to provide support, as needed (e.g., suggesting the participant write at home rather than in the ICU [a potentially less chaotic environment], encouraging use of key words or focusing on the present if events are hard to summarize).

### Control

Participants randomized to the control group received standard contact and communication from ICU/hospital doctors and were contacted by the study team only to complete study questionnaires. As part of CANOE-F, all participants (intervention and control) completed a baseline questionnaire at patient bedside, an interview prior to patient discharge, and an assessment one month after patient discharge from the hospital.

### Measures

Table 1 summarizes study measures (excluding demographic variables), the timepoints at which each measure was captured, and the method of assessment (see S2 Appendix for all survey items in English and Spanish). No changes to measures occurred after the trial commenced. The study team had access to information that could identify participants during and after data collection.

**Table 1:** Details regarding study measures, assessment method, timepoint at which it was assessed, and measure type.

Measure	Assessment Method	Timepoint	Measure Type
Recruitment	% of eligible participants accrued in study	Baseline	Primary outcome
Retention	% of accrued participants remaining in study	30-days after end of hospital care	Primary outcome
Compliance with intervention	% of intervention participants who reported writing in the ICU diary ≥ 2 times per week during hospital care	30-days after end of hospital care	Secondary outcome
Compliance with assessments	% of accrued participants completing at least 90% of survey assessments	30-days after end of hospital care	Secondary outcome
Acceptability	% of intervention participants with a score ≥ 4 on the 4-item Acceptability of Intervention Measure [31]	30-days after end of hospital care	Secondary outcome
Feasibility	% of intervention participants with a score ≥ 4 on the 4-item Feasibility of Intervention Measure [31]	30-days after end of hospital care	Secondary outcome
Appropriateness	% of intervention participants with a score ≥ 4 on the 4-item Intervention Appropriateness Measure [31]	30-days after end of hospital care	Secondary outcome
Cardiac fear	8-item fear subscale of the Cardiac Anxiety Questionnaire [32]	End of hospital care; 30-days after end of hospital care	Additional outcome
Cardiac avoidance	5-item avoidance subscale of the Cardiac Anxiety Questionnaire [32]	End of hospital care; 30-days after end of hospital care	Additional outcome
Posttraumatic stress symptoms	20-item PTSD Checklist for DSM-5 [33]	30-days after end of hospital care	Additional outcome

#### Primary outcomes

*Recruitment*. Recruitment success was assessed by calculating the number who provided verbal consent to participate in the study as a proportion of those referred to the study.

*Retention*. Retention success was assessed by calculating the proportion of participants who remained in the study at follow-up out of the total number enrolled.

#### Secondary outcomes

*Compliance*. Compliance with the study protocol was calculated as the proportion of participants completing at least 90% of all study assessments (i.e., 90% non-missing questionnaire items). Compliance with the intervention was calculated as the proportion of intervention participants who reported writing in the diary at least 2 times per week while their loved one was in the hospital.

*Acceptability*. Acceptability was self-reported by intervention participants at the follow-up session. Acceptability was assessed using the 4-item Acceptability of Intervention Measure (AIM; e.g., “I like the ICU diary”) [31]. Each item is measured on a scale from 1, *Completely disagree*, to 5, *Completely agree*, and scores are computed by taking the mean of all items (*Cronbach’s α* = 0.94). A mean score of 4 (*Agree*) or greater was pre-specified as the cutoff for indicating agreement that the intervention was acceptable.

*Feasibility*. Feasibility was self-reported by intervention participants at the follow-up session and was assessed using the 4-item Feasibility of Intervention Measure (FIM; e.g., “The ICU diary seems easy to use”) [31]. Each item is measured on a scale from 1, *Completely disagree*, to 5, *Completely agree*, and scores are computed by taking the mean of all items (*Cronbach’s α* = 0.82). A mean score of 4 (*Agree*) or greater was pre-specified as the cutoff for indicating agreement that the intervention was feasible.

*Appropriateness*. Appropriateness was self-reported by intervention participants at the follow-up session and was assessed using the 4-item Intervention Appropriateness Measure (IAM; e.g., “The ICU diary seems suitable for reducing fear about my loved one’s heart”) [31]. Each item is measured on a scale from 1, *Completely disagree*, to 5, *Completely agree*, and scores are computed by taking the mean of all items (*Cronbach’s α* = 0.93). A mean score of 4 (*Agree*) or greater was pre-specified as the cutoff for indicating agreement that the intervention was appropriate.

*Participant feedback*. Additional feedback about the acceptability of the intervention was gathered during the follow-up session using 5 open-ended questions. Specifically, these participants were invited to share things that they liked about the ICU diary, any challenges, advice for writing an ICU diary, and what they would like to change about the ICU diary. They were also given the opportunity to share anything else about their experience writing in the diary, if desired, and asked to report how frequently they wrote in the diary each week during and after their loved one’s ICU stay.

#### Exploratory outcomes

*Cardiac fear*. At the post-intervention session and at the follow-up session, participants self-reported cardiac fears using the 8-item fear subscale of the Cardiac Anxiety Questionnaire (CAQ) [32], which assesses fears about the condition of the patient’s heart. Items were modified to ask about the patient’s heart (e.g., “If my loved one’s tests come out normal, I still worry about their heart,” “When my loved one has chest discomfort, or when their heart is beating fast: … I get frightened”). Items were scored using a 5-point Likert scale ranging from 1, *Never*, to 5, *Always*. This scale is scored by taking the mean of all items. At end of hospital care, *Cronbach’s α* = 0.79; at follow-up, *Cronbach’s α* = 0.74.

*Cardiac avoidance*. At the post-intervention session and at the follow-up session, participants self-reported cardiac avoidance about the patient’s heart using the 5-item avoidance subscale of the CAQ [32], which assesses avoidance of activities that may raise the patient’s heart rate. Items were modified to ask about the patient’s heart (e.g., “My loved one should avoid physical exertion”). Items were scored using a 5-point Likert scale ranging from 1, *Never*, to 5, *Always*. This scale is scored by taking the mean of all items. At end of hospital care, *Cronbach’s α* = 0.88; at follow-up, *Cronbach’s α* = 0.88.

*PTSS*. Participants self-reported the presence, frequency, and severity of PTSS symptoms at the follow-up session in relation to either “The heart problem/symptoms your loved one experienced that brought you to the hospital,” “The experience in the Emergency Department,” “The experience in the ICU,” or “The experience after the ICU” using the 20-item PTSD Checklist for DSM-5 (PCL-5) [33]. The PCL-5 corresponds to DSM-V criteria and assesses PTSS over the past month using a 5-point Likert scale ranging from 0, *Not at all*, to 4, *Extremely*. This scale is scored by summing all items, with scores ≥ 33 indicating clinically significant PTSS [34]. This scale is valid and reliable for use in medical populations [35]. Internal consistency reliability was high, *Cronbach’s α* = 0.91.

#### Demographic variables

During the baseline/pre-intervention session, participants self-reported biological sex, gender, age, sexual orientation, race/ethnicity, education, income, relationship type to patient (e.g., child, partner/spouse), relationship length, living situation (type of residence, whether they live with the patient), presence during the patient’s CA event, insurance status, and primary language (English or Spanish).

#### Intervention fidelity

An intervention fidelity checklist was implemented to ensure that all components of the intervention were delivered completely and with consistency for the baseline session, at which the ICU diary instructions were provided, and for weekly check-ins, as well as to ensure that no control participants received any intervention components.

#### Changes to study protocol

Four notable changes occurred throughout the course of the study. First, the protocol was amended to include Spanish speakers as eligible participants. Second, although the protocol initially only included cohabiting partners/spouses of patients, this was expanded to include non-partner family members to facilitate recruitment. Third, the target enrollment was increased to recruit one additional participant in the diary intervention because one person who was randomized to the diary intervention dropped out immediately after randomization. This resulted in a total enrollment of 16 participants (11 in the diary intervention, 5 in the control group) rather than 15 participants as originally intended. Fourth, participants were originally compensated $25 for survey completion at the end of hospital care and $25 for survey completion 30 days after the end of hospital care. This was later amended to be one $50 payment at 30 days after the end of hospital care, such that, if a participant’s loved one passed away, they were not contacted immediately following the death and were not monetarily penalized for missing this assessment.

#### Data analysis strategy

Participant data were analyzed as randomized. For the primary outcomes of recruitment and retention at 30 days after the end of hospital care, as well as secondary outcomes of compliance with the intervention (write ≥ 2 times per week), compliance with study procedures (complete ≥ 90% of study assessments), and appropriateness, feasibility, and acceptability benchmarks (mean scale score ≥ 4), we computed the proportion of participants meeting these criteria and 95% confidence intervals around these proportions. Descriptive statistics for continuous appropriateness, feasibility, and acceptability scores are also reported.

For additional outcomes of cardiac fear and cardiac avoidance, both at the end of hospital care as well as at 30 days after the end of hospital care, and for PTSS at 30 days after the end of hospital care, we conducted independent samples t-tests to compare scores in the intervention (diary) v. control group. Nonparametric Wilcoxon Rank Sum tests were conducted as sensitivity analyses. For PTSS, we additionally Chi-Square tests comparing proportion of intervention (diary) v. control group participants with a positive screen for clinically significant PTSS (scale score ≥ 33). All statistical tests for additional outcomes are considered exploratory and primarily descriptive because the FAID Fear pilot study was not powered to detect significant differences. Qualitative responses for intervention feedback are also described.

Explicit permission from participants to post de-identified data online was not obtained; rather, participants consented that, “Upon removal of identifiers from the dataset, data may be shared with other researchers.” In accordance with recommendations for sharing raw clinical data for publication stating that it is ideal to obtain this consent [36], data are available only by request from the Roybal center at Columbia (please contact Alexandra M. Miecznikowski by email: as5068@cumc.columbia.edu).

#### Sample size determination

Sample size was guided by the need to enroll enough participants to examine the feasibility of recruiting, retaining, and assessing participants, and of implementing the FAID Fear intervention with good compliance in this population to inform the design of a fully powered RCT. Assuming retention rates of ~90%, a sample size of 15 participants would allow us to compute a 95% confidence interval around this estimate of 75%– 100%, providing sufficient precision for planning a larger trial. This study was not powered to detect significant effects of the intervention on mechanistic outcomes (i.e., cardiac fear, PTSS).

## Results

CONSORT guidelines for randomized pilot and feasibility trials [37] were followed in reporting study results. Enrollment occurred between November 29, 2021 –September 30, 2022, and follow-up sessions were conducted between January 21, 2022 –November 30, 2022. The trial ended when recruitment goals were met. Of the 15 family members who completed the baseline assessment and provided demographic information, most were partners (10; 66.7%) and had been in a relationship with the patient for 20.70 years on average (*SD* = 13.25; *Range* = 4.0, 43.0). Thirteen family members reported that they lived with the patient (86.7%). Full demographic information is detailed in Table 2.

**Table 2:** Demographic information overall and stratified by intervention v. control participants.

		Overall (*N* = 15)	Intervention (*n* = 10)	Control (*n* = 5)
		Mean (*SD*) or N (%)	Mean (*SD*) or N (%)	Mean (*SD*) or N (%)
Age		50.73 (13.41)	51.50 (13.32)	49.20 (15.04)
Sex	*Female*	12 (80.0%)	9 (90.0%)	3 (60.0%)
	*Male*	3 (20.0%)	1 (10.0%)	2 (40.0%)
Gender	*Woman*	12 (80.0%)	9 (90.0%)	3 (60.0%)
	*Man*	3 (20.0%)	1 (10.0%)	2 (40.0%)
Relationship to patient	*Partner*	10 (66.7%)	7 (70.0%)	3 (60.0%)
	*Child*	3 (20.0%)	2 (20.0%)	1 (20.0%)
	*Parent*	1 (6.7%)	1 (10.0%)	0 (0.0%)
	*Sibling*	1 (6.7%)	0 (0.0%)	1 (20.0%)
Live with patient	*Yes*	13 (86.7%)	9 (90.0%)	4 (80.0%)
Relationship length (years)^a^		20.70 (13.25)	19.86 (11.77)	22.67 (19.14)
Race	*Black/African American*	4 (26.7%)	3 (30.0%)	1 (20.0%)
	*White*	9 (60.0%)	6 (60.0%)	3 (60.0%)
	*Other*	2 (13.3%)	1 (10.0%)	1 (20.0%)
Ethnicity	*Hispanic or Latino*	5 (33.3%)	2 (20.0%)	3 (60.0%)
Language	*English*	12 (80.0%)	2 (20.0%)	1 (20.0%)
	*Spanish*	3 (20.0%)	8 (80.0%)	4 (80.0%)
Health Insurance^b^	*Yes*	13 (86.7%)	8 (80.0%)	5 (100.0%)
Education	*High School/GED*	4 (26.7%)	2 (20.0%)	2 (40.0%)
	*Some College*	3 (20.0%)	2 (20.0%)	1 (20.0%)
	*Associate’s Degree*	1 (6.7%)	1 (10.0%)	0 (0.0%)
	*College Degree*	4 (26.7%)	3 (30.0%)	1 (20.0%)
	*Graduate/ Professional Degree*	3 (20.0%)	2 (20.0%)	1 (20.0%)
Income^c^	*$0 - $9*,*999*	0 (0.0%)	0 (0.0%)	0 (0.0%)
	*$10*,*000 –$24*,*999*	2 (28.6%)	2 (33.3%)	0 (0.0%)
	*$25*,*000 –$49*,*999*	3 (42.9%)	2 (33.3%)	1 (100.0%)
	*$50*,*000 –$99*,*999*	0 (0.0%)	0 (0.0%)	0 (0.0%)
	*$100*,*000 –$149*,*999*	1 (14.3%)	1 (16.7%)	0 (0.0%)
	*$150*,*000 or more*	1 (14.3%)	1 (16.7%)	0 (0.0%)

^a^Partners of CA patients only (*n* = 10; 7 in the diary intervention, 3 in the control group)

^b^Health insurance over the past 2 years

^c^This was not initially queried; only *n* = 9 participants were asked this question (2 declined to respond)

Slightly more than half of the family member participants were not present at any point during their loved one’s CA (*n* = 8; 53.3%), the remaining 7 either witnessed the CA, found the patient, were present when resuscitation measures were instituted, and/or were present during some other aspect of the CA (46.7%).

Assessments at the end of hospital care occurred a median of 16.5 days after enrollment into the study (*Mean* = 27.79; *SD* = 29.82; *Range* = 6, 120), and assessments at 30 days after the end of hospital care occurred a median of 46 days after enrollment (*Mean* = 55.21; *SD* = 29.52; *Range* = 34, 151). No harms or unintended effects occurred during the study. Procedures were implemented with 100% fidelity.

### Primary outcomes

**Fig 1:**
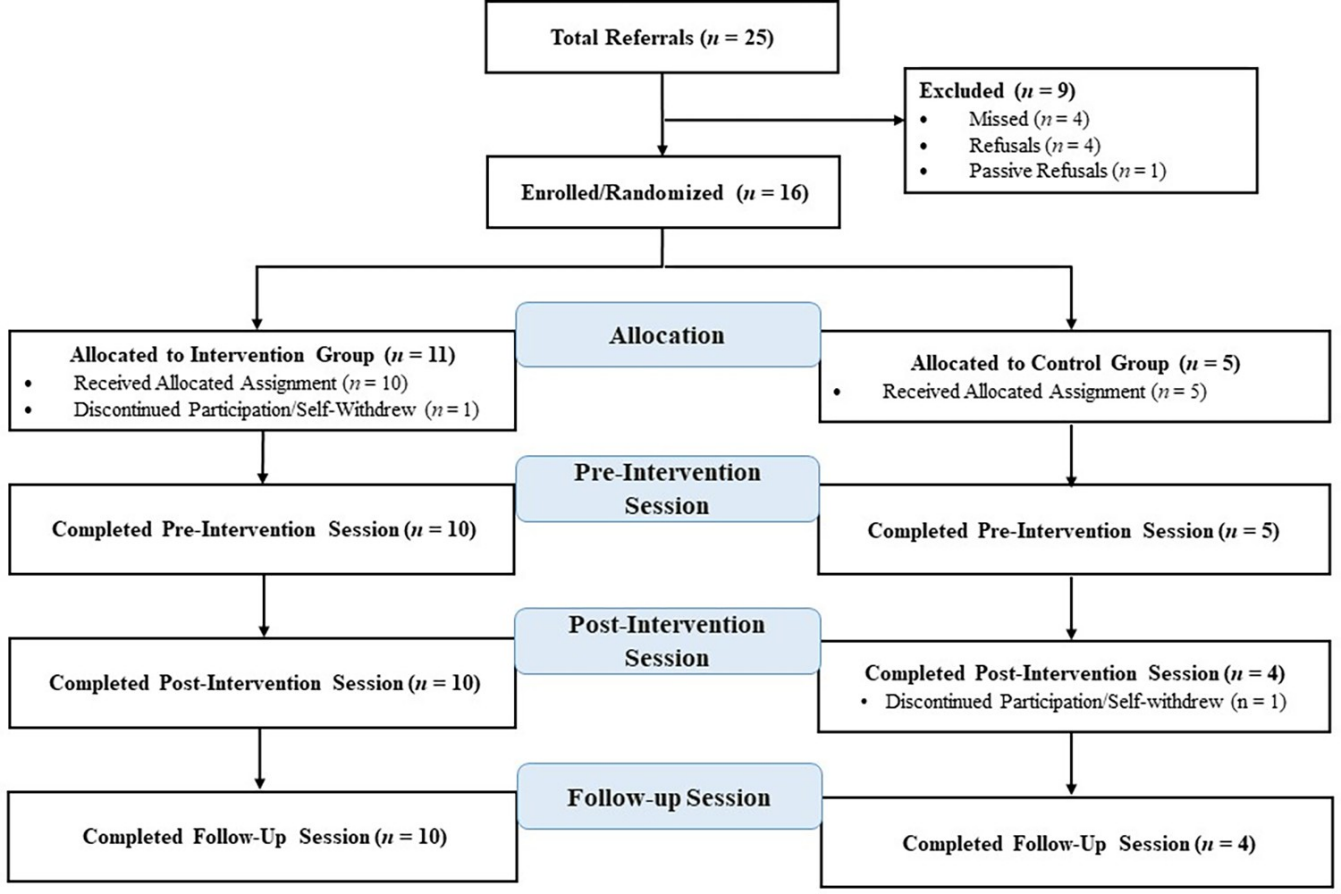
Study CONSORT flow diagram.

The study CONSORT Flow Diagram is in Fig 1. Of the 25 potential participants referred to study staff, 16 (64.0%) of these provided informed consent and enrolled in the pilot study (95% Confidence Interval [CI] 42.5%, 82.0%). Four of these 25 declined to participate (16.0%) and 5 were unable to be reached (20.0%). Of the 16 enrolled participants, most (*n* = 14; 87.5%) completed the final assessment 30 days after the end of hospital care (95% CI 61.7%, 98.5%), with 10 out of 11 (90.9%) retained in the diary intervention and 4 out of 5 (80.0%) retained in the control group.

### Secondary outcomes

Fourteen participants provided complete data for at least 90% of survey assessments (87.5%; 95% CI 61.7%, 98.5%). Of the 11 participants randomized to the diary intervention, 7 reported that they wrote in the diary at least twice a week up until the end of hospital care (63.6%; 95% CI 30.8%, 89.1%). For the 10 diary intervention participants who completed the study, this was 70.0% (95% CI 34.8%, 93.3%). The number of days per week participants reported writing in their diaries during hospital care ranged from 0 to 7 days (*Median* = 3 days).

At 30 days after the end of hospital care, of the 10 participants remaining in the diary intervention, 7 were classified as agreeing that the ICU diary intervention was appropriate for reducing cardiac fears about their loved one’s heart (70.0%; 95% CI 34.8%, 93.3%; *Mean* = 3.85; *SD* = 0.95; *Median* = 4.13; *Range* = 2.00, 5.00), 9 were classified as agreeing that the ICU diary intervention was feasible (90.0%; 95% CI 55.5%, 99.8%; *Mean* = 4.18; *SD* = 0.46; *Median* = 4.13; *Range* = 3.25, 5.00), and 8 were classified as agreeing that the ICU diary intervention was acceptable (80.0%; 95% CI 44.4%, 97.5%; *Mean* = 4.20; *SD* = 0.94; *Median* = 4.38; *Range* = 2.50, 5.00).

Feedback regarding the diary intervention was overwhelmingly positive. Nine participants stated that they liked the diary or that it helped them to process things, express themselves, or feel less alone (90%). Participants advised others who might complete an ICU diary to use it as an outlet to express themselves and reflect on their experiences or shared that it helped them to “escape” or to help to free themselves from the pain they are feeling. In terms of challenges, one participant stated that it was hard to write when the health of their loved one was uncertain, another struggled to find time to write, and a third shared that it was difficult to focus on the diary once the patient had returned home. Of note, no participants suggested any changes to the diary protocol.

### Additional outcomes

Descriptive statistics for additional outcomes are in Table 3. There was no significant difference in family members’ cardiac fear (CAQ fear subscale) between intervention v. control participants at the end of hospital care (*Mean Δ* = 0.24, 95% CI -0.83, 1.32, *p* = .63) or at 30 days after the end of hospital care (*Mean Δ* = 0.08, 95% CI -0.92, 1.08, *p* = .86; nonparametric Wilcoxon Mann Whitney tests were also nonsignificant, (*χ*^*2*^(1) = 0.18, *p* = .67), and, (*χ*^*2*^(1) = 0.02, *p* = .89), respectively). In addition, there was no significant difference in family members’ cardiac avoidance (CAQ avoidance subscale) between intervention v. control participants at the end of hospital care (*Mean Δ* = -0.11, 95% CI -1.43, 1.21, *p* = .86) or 30 days after the end of hospital care (*Mean Δ* = 0.19, 95% CI -1.10, 1.48, *p* = .75; nonparametric Wilcoxon Mann Whitney tests were also nonsignificant, (*χ*^*2*^(1) = 0.25, *p* = .62), and, (*χ*^*2*^(1) = 0.08, *p* = .78), respectively).

**Table 3:** Descriptive statistics for additional outcomes at end of hospital care and 30-days after the end of hospital care stratified by intervention v. control participants.

			Intervention (*n* = 10)	Control (*n* = 4)^a^
End of Hospital Care	Cardiac Fear	*Mean (SD)*	3.35 (0.90)	3.59 (0.61)
		*Range*	3.00, 4.38	3.00, 4.38
	Cardiac Avoidance	*Mean (SD)*	3.56 (1.14)	3.45 (0.53)
		*Range*	1.40, 5.00	3.00, 4.20
30-Days after End of Hospital Care	Cardiac Fear	*Mean (SD)*	3.33 (0.88)	3.41 (0.33)
		*Range*	1.50, 4.50	3.13, 3.75
	Cardiac Avoidance	*Mean (SD)*	3.16 (1.07)	3.35 (0.72)
		*Range*	1.60, 5.00	2.80, 4.40
	PTSS^a^	*Mean (SD)*	19.70 (12.83)	11.00 (4.08)
		*Median*	18.00	11.00
		*Range*	6.00, 43.00	7.00, 15.00
	Positive PTSS screen^b^	*N (%)*	2 (20.0%)	0 (0.0%)

^a^*n* = 1 participant in the control group did not complete assessments at the end of hospital care or 30-days after the end of hospital care

^b^Posttraumatic stress symptoms

^c^Score ≥ 33

At 30 days after the end of hospital care, there was no significant difference in PTSS (PCL-5) between intervention v. control participants (*Mean Δ* = -8.70, 95% CI -23.26, 5.86, *p* = .22). A nonparametric Wilcoxon Mann Whitney test also resulted in a nonsignificant difference between intervention v. control participants (*χ*^*2*^(1) = 1.29, *p* = .26), as did an analysis examining positive screen for clinically significant PTSS (score ≥ 33; *Fisher’s Exact Test*, *p* = 1.00).

## Discussion

The FAID Fear pilot RCT demonstrated the feasibility of conducting a larger RCT evaluating a theory-based and mechanism-focused family-authored ICU diary intervention to reduce distress in patients experiencing CA. Primary outcomes demonstrated excellent recruitment and retention rates, ascertainment of primary outcomes, and adherence to the diary intervention. Participant reports of feasibility, acceptability, and appropriateness of the diary intervention for reducing fear about their loved one’s heart were also promising.

Given such promising initial indicators of feasibility, the FAID Fear intervention is well-suited to being scaled up and implemented in a larger study powered to test mechanism engagement (i.e., cardiac fear, PTSS). For example, in the study by Nielsen et al., on which our protocol was based [30, 38], nearly as many family members declined participation as enrolled (*n* = 409 assessed for eligibility; 114 declined, 116 enrolled). Because potential participants referred to the FAID Fear study team already met preliminary eligibility criteria and had expressed a willingness to be contacted about future research, our recruitment rate of nearly two-thirds is not directly comparable, but it does increase confidence in our ability to meet target enrollment goals. The fact that all but two of the family members who consented to participate in the study and were randomized completed all study assessments and provided complete data is also compatible with previously successful large-scale RCTs testing the efficacy of family-authored ICU diary interventions [30, 38].

In general, family members in the diary intervention agreed that the diary was appropriate, feasible, and acceptable. Even though participants were instructed to write as often as they want, “preferably at least twice a week,” most met this self-reported minimum, and many wrote even more than suggested. Some shared that they found the diary so beneficial that they continued their use of the diary even after the intervention period ended. Some participants also experienced a delay in receiving the diary after completing the baseline session, affecting intervention engagement. Because these recommendations were not an explicit rule, it may be that strengthening this language would result in even greater adherence to the diary intervention. Future studies should also consider the optimal frequency of writing in the ICU diary.

Due to its mechanistic focus on the potential social transmission of distress from family members to patients experiencing CA, it is worth noting that cardiac fear was lower for participants in the diary intervention (v. the control group) at the end of hospital care, and both cardiac fear and cardiac avoidance were lower for participants in the diary intervention (v. the control group) at 30 days after the end of hospital care. Although underpowered, this provides preliminary evidence that cardiac fear and cardiac avoidance in family members may serve as a proximal target mechanism for improving patient outcomes (or, at minimum, provides no indication to the contrary). Indeed, increased attention has been drawn to the essential role of close others in determining health outcomes and the promise of socially-based interventions to improve health and well-being [39–41]. Family member’s PTSS stand in contrast to the findings for cardiac fear and cardiac avoidance, however. We suspect that this trend is due to chance, since no previous RCTs have found significantly greater PTSS in family members receiving an ICU study [24, 25]. The one large study included in a 2019 meta-analysis [25] of family-authored ICU diaries that did find higher PTSS in family members randomized to the diary intervention (*n* = 281) v. the control group (*n* = 282) was also nonsignificant [42]. Some controversy exists regrading psychological debriefing immediately after a traumatic event, with limited evidence for effectiveness and potential evidence of harm [43]. ICU diaries differ from psychological debriefing in that they are guided by the family member themselves, allowing for more natural and self-directed engagement in processing the traumatic CA. This is somewhat similar to brief written exposure therapy, a promising treatment for PTSD in which individuals write about their traumatic experience across five sessions to process their emotions and engage in mean-making [44]. It is encouraging that no intervention participants stated that the diary increased their distress, and this study was not powered to detect a significant effect of the intervention on PTSS. Still, careful oversight of adverse events and the inclusion of a trained clinical psychologist in future studies is warranted.

FAID Fear is unique among ICU diary interventions in that the diaries are intended for family members and not for survivors. It was not known whether this intervention would be feasible in a population that often focuses solely on the health and well-being of their loved one, who has experienced a CA, at the expense of their own needs [27, 28]. Yet, a high degree of success was achieved. Not only do interventions targeted towards the dyad result in more positive outcomes than those that target patients alone [45, 46], the emphasis on understanding theory-informed [15] socially-based mechanisms of change is also innovative, timely [39–41], and critical to support efforts to increase the potency of interventions and disseminate them widely [47, 48]. Furthermore, should a fully powered RCT provide evidence for efficacy, FAID Fear was designed to have high potential for scalability given the low burden on study and medical staff, the low cost, and minimal privacy concerns (e.g., the diary is written only by the family member and is not part of the medical record) [30]. The FAID Fear diary is different from prior interventions in the sense that the participant is not asked to share their writing with anyone. It is worth noting that we asked participants if they would be willing to share their diary with the study (to gauge feasibility for future studies, e.g., for coding diary content), and all but one participant replied that they would be.

Study successes must be considered in light of several limitations. First and foremost, given the primacy of romantic relationships for shaping health and well-being [39–41], the original intention was to recruit romantic relationship partners only (rather than family members broadly). Indeed, the theory informing the design of this study also focuses on relationship partners [15]. This change was made to facilitate meeting our recruitment goals. If a focus on partner relationships is maintained, larger trials may have to include multiple study sites to meet recruitment goals. It is also possible that this expansion would not have been necessary were participants not drawn from CANOE-F, which enrolled any family member regardless of relationship type (i.e., partner relationships were not prioritized). Thus, if a patient did have a primary partner who may have otherwise been interested in FAID Fear but a child or other relative was enrolled in CANOE-F, this partner would have been missed by our study team. A larger RCT that is not tied to the recruitment structure of another study will be better equipped to focus solely on partner relationships. That said, social transmission of fear can occur within all close relationships, and we expect that any family member taking on the primary caregiving role would contribute significantly to patients’ well-being after a CA [15, 18, 23]. Recruiting from CANOE-F may have biased our sample in that they were only referred if they expressed interest in hearing about future studies, so results for recruitment and retention rates may not generalize to studies in which family members are directly recruited (see S3 Appendix for a flow diagram of CANOE-F recruitment rates and referrals). These participants may also have been more likely to find the study feasible, acceptable, and appropriate. Similarly, providing a financial incentive may have resulted in bias due to a desire to remain in the study and receive compensation. However, participation and compensation were not contingent on completing the diary. Results may also not generalize to other hospital settings (e.g., non-urban).

The Cardiac Anxiety Questionnaire (CAQ) is validated for use when asking about a person’s own health [32] and was modified for this study to query family members’ cardiac fear and cardiac avoidance regarding the CA survivor. These assessments were additional outcomes (i.e., not primary or secondary) and are face valid. Furthermore, research shows that family members report continuing fear about the survivor’s heart and possible recurrence up to six months after the CA [27] and family members of ICU patients have elevated scores on the CAQ as compared to the general population [6]. Still, further validation studies will be necessary for larger studies using these measures as primary endpoints. The CAQ has also not yet been directly linked to outcomes after CA. However, research in other cardiac populations has found that the fear and avoidance subscales of the CAQ are associated with worse physical health [2, 49]. The association of the CAQ with CA survivors’ outcomes is currently under investigation. Some participants did not receive the diary right away due to delays in mailing (e.g., lost packages), and, due to privacy concerns, diaries were not collected to confirm adherence to recommended frequency of writing. Future studies should consider collecting diaries to confirm frequency of writing. Such studies might also note the length of diary entries and could include qualitative analysis of diary content. Due to the study design, participants and study staff were not blind to random assignment. In a larger RCT, it will be possible to blind study staff conducting survey assessments and data analysts. Participants cannot be blinded; however, it is possible to track contamination by asking whether control participants kept a diary. No control participants opted to do so in this pilot study. Finally, patients were not targeted for recruitment in this pilot study, a critical consideration for designing dyadic studies to test how family members impact patient outcomes.

## Conclusions

FAID Fear is timely, innovative, and multidisciplinary in scope. This pilot study was designed to build from basic behavioral science (i.e., problem characterization, identification of modifiable intervention targets) to the development of theory-based survivor/family-targeted dyadic interventions that can be implemented in the ICU.

Given the uncertainty that family members of CA patients in the ICU would be able to find the time for, let alone freely partake in, a diary intervention intended to benefit their well-being while their loved one’s recovery and health status remains uncertain, this pilot study showed preliminary evidence that ICU diary interventions are not only feasible but acceptable and appropriate for family members of patients experiencing CA. Our high levels of enrollment and retention, as well as high engagement and acceptability by family members for the diary intervention, are encouraging indicators that a more definitive study powered to detect efficacy outcomes in family members, and pilot recruitment procedures in family member/CA survivor dyads, should be conducted.

## Supporting information

S1 Appendix ICU diary instructions in English and Spanish. (PDF)

S2 Appendix Survey assessments in English and Spanish. (DOCX)

S3 Appendix CONSORT diagram for CANOE-F. (JPG)
