# Supplementary material for: Family-authored ICU diaries to reduce fear in patients experiencing a cardiac arrest (FAID fear): A pilot randomized controlled trial
Source: PLoS One. 2023 Jul 27;18(7):e0288436. doi: 10.1371/journal.pone.0288436 (PMC10373992; doi:10.1371/journal.pone.0288436)
Supplement: S1 Appendix — (PDF) [file pone.0288436.s001.pdf]

APPENDIX A: Diary Instructions for Participants (English Version)

ICU Diaries:
A Guide to Writing About Your Experience

A stay on an Intensive Care Unit (ICU) can be frightening and upsetting for patients' families and friends. Writing about the ICU experience in a diary has helped others to cope during this difficult time. We believe that writing an ICU diary may help you to cope as well.

What is a cardiac arrest?

Cardiac arrest is a life-threatening emergency in which the heart suddenly stops beating due to a problem with the electricity in the heart. When this happens, the heart cannot pump blood to the brain, lungs, and other organs. This causes a person with cardiac arrest to collapse and stop responding.

Cardiac arrest leads to death in minutes if help is not provided right away. People who survive an initial cardiac arrest often go on to receive care in a Cardiac or Neurologic ICU. Many patients will be kept asleep medically to give the body a chance to recover. Because of the lack of oxygen to the brain that occurs during the cardiac arrest, many patients will experience long-term effects such as loss of memory, fatigue, and dizziness. Many patients will also not remember the events around the time of the cardiac arrest.

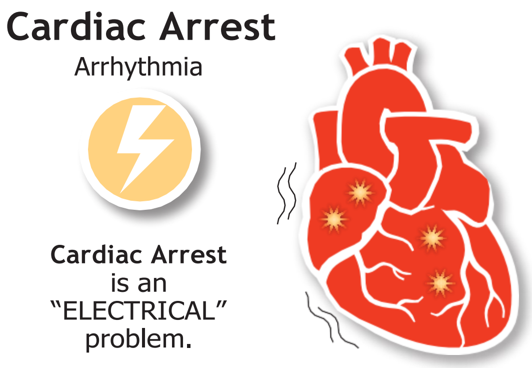

Why write an ICU Diary?

The aim of writing an ICU diary is to help you process what happens to you and to your loved one to help you cope during this stressful and difficult period. It may also help you make sense of what happened and move forward in life after intensive care. Some potential topics to write about are included on the next page.

Patients may also find an ICU diary beneficial. Patients who leave the ICU may have no memories of the cardiac arrest and the time in the hospital. Other patients can leave the ICU with frightening memories of their time in the ICU. You and your loved one can read the diary together during recovery and understand what happened.

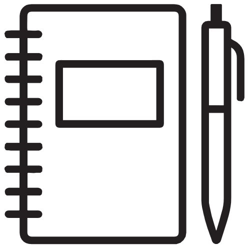

APPENDIX A: Diary Instructions for Participants (English Version)

What can you write about?

The beginning

- What happened when your loved one fell ill?
- How did your loved one get to the ICU?
- What did you do? What did you think and feel?

The time in the ICU

- What is the condition of your loved one?
- What happens to your loved one (e.g., medical activities, visits)?
- What information do you and your loved one receive?
- How do you react to treatment, information, care activities, and visits? How does your loved one react?
- What are some recovery milestones, like first opening the eyes, standing or sitting in a chair, coming off the ventilator, etc.?
- What happens in your loved one's life, in the family, and in society, that they may find interesting (e.g., what do grandchildren, friends, or pets do; how is the home)?
- How do you think and feel about the situation?

After discharge from the ICU

- How does your loved one improve?
- What happens in your loved one's life, in the family, and in society, that they may find interesting (e.g., what do grandchildren, friends, or pets do; how is the home)?
- What are your feelings, worries, and fears?

How often/how much should you write?

Writing often may help you feel connected. Write as often as you would like, preferably at least twice a week. These entries can be as long or as short as you would like.

Sharing the diary with your loved one

You can share your diary with your loved one if you choose.

The diary may be difficult for your loved one to read. They may have a hard time concentrating, or they may not want to learn about the time they spent in the ICU. You should not push your loved one to read the diary before they are ready.

Your loved one may like for you to read aloud from the diary so that you can talk about what happened together. Talking about the difficult time in the ICU may help you both to recover.

Contact information

If you have any questions, please ask the research staff or the providers in the ICU.

Our research staff can be reached here:

Talea Cornelius
tmc2184@cumc.columbia.edu

Sachin Agarwal
sa2512@cumc.columbia.edu

All the best for you and your loved one!

APPENDIX A: Diary Instructions for Participants (Spanish Version)

Diarios de la UCI:

Una Guía Para Escribir Sobre Su Experiencia

Una estadía en una Unidad de Cuidados Intensivos (UCI) puede ser aterradora y una molestia para los familiares y amigos de los pacientes. Escribir sobre la experiencia de la UCI en un diario ha ayudado a otras personas a sobrellevar este momento difícil. Creemos que escribir en un diario de la UCI también puede ayudarlo a sobrellevar la situación.

¿Qué es un paro cardíaco?

Un paro cardíaco es una emergencia potencialmente mortal en la que el corazón de repente deja de latir debido a un problema con la corriente eléctrica del corazón. Cuando esto sucede, el corazón no puede bombear sangre al cerebro, los pulmones y otros órganos. Esto hace que una persona que tiene un paro cardíaco se desmaye y deje de responder.

Un paro cardíaco conduce a la muerte en minutos si no se recibe ayuda de inmediato. Las personas que sobreviven a un paro cardíaco a menudo reciben atención medica en una UCI cardíaca o neurológica. A muchos pacientes se les mantendrá dormidos médicamente para que el cuerpo tenga la oportunidad de recuperarse. Debido a la falta de oxígeno en el cerebro que ocurre durante un paro cardíaco, muchos pacientes tendrán efectos a largo plazo como pérdida de memoria, fatiga y mareos. Muchos pacientes tampoco recordarán los eventos que ocurrieron en el momento del paro cardíaco.

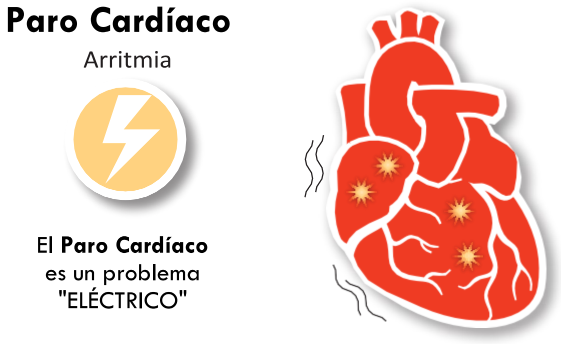

¿Por qué escribir un diario de UCI?

El objetivo de escribir un diario de la UCI es para ayudarlo a procesar lo que le sucede a usted y a su ser querido, y así ayudarlo a sobrellevar este período estresante y difícil. También puede ayudarlo a entender lo que sucedió y seguir adelante en la vida después de los cuidados intensivos. En la siguiente página se incluyen algunos temas sobre los que puede escribir.

Los pacientes también pueden encontrar beneficioso un diario de la UCI. Los pacientes que abandonan la UCI pueden no tener recuerdos del paro cardíaco y del tiempo en el hospital. Otros pacientes pueden salir de la UCI con recuerdos aterradores de su tiempo en la UCI. Usted y su ser querido pueden leer el diario juntos durante la recuperación y comprender lo que sucedió.

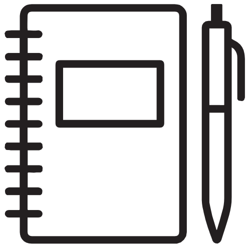

APPENDIX A: Diary Instructions for Participants (Spanish Version)

¿Sobre qué puede escribir?

El principio

- ¿Qué sucedió cuando su ser querido se enfermó?
- ¿Cómo llegó su ser querido a la UCI?
- ¿Qué hizo usted? ¿Qué pensó y sintió usted?

El tiempo en la UCI

- ¿Cuál es la condición de su ser querido? (¿Cómo se encuentra su ser querido?)
- ¿Qué le sucede a su ser querido (por ejemplo, actividades médicas, visitas)?
- ¿Qué información reciben usted y su ser querido?
- ¿Cómo reacciona usted al tratamiento, la información, las actividades de cuidado y las visitas? ¿Cómo reacciona su ser querido?
- ¿Cuáles son algunos de los momentos más importantes de la recuperación, como abrir los ojos por primera vez, ponerse de pie o sentarse en una silla, ser desconectado del respirador, etc.?
- ¿Qué sucede en la vida de su ser querido, en la familia y en la sociedad, que le resulta interesante a su ser querido (por ejemplo, qué hacen los nietos, los amigos o las mascotas; qué pasa en su casa)?
- ¿Cómo piensa y se siente usted acerca de la situación?

Después del alta de la UCI

- ¿Cómo mejora su ser querido?
- ¿Qué sucede en la vida de su ser querido, en la familia y en la sociedad, que le resulta interesante a su ser querido (por ejemplo, qué hacen los nietos, los amigos o las mascotas; qué pasa en su casa)?
- ¿Cuáles son los sentimientos de usted, sus preocupaciones y sus miedos?

¿Con qué frecuencia / cuánto debería escribir?

Escribir con frecuencia puede ayudarlo a sentirse conectado. Escriba con la frecuencia que usted desee, preferiblemente al menos dos veces por semana. Puede escribir lo que quiera, una frase corta o algo mucho más largo.

Compartir el diario con su ser querido

Puede compartir su diario con su ser querido si usted lo desea.

El diario puede ser difícil de leer para su ser querido. Es posible que su ser querido tenga dificultad para concentrarse o que no quiera saber sobre el tiempo que pasó en la UCI. Usted no debe presionar a su ser querido para que lea el diario antes de que su ser querido esté listo.

Es posible que a su ser querido le guste que usted lea en voz alta el diario para que puedan hablar sobre lo que sucedió juntos. Hablar sobre el momento difícil en la UCI puede ayudarlos a ambos a recuperarse.

Información del contacto

Si tiene alguna pregunta, consulte al personal del estudio o a los proveedores de la UCI.

Puede comunicarse con nuestro personal del estudio:

Talea Cornelius
tmc2184@cumc.columbia.edu

Sachin Agarwal
sa2512@cumc.columbia.edu

¡Todos los mejores deseos para usted y para su ser querido!
