# Supplementary material for: Family-authored ICU diaries to reduce fear in patients experiencing a cardiac arrest (FAID fear): A pilot randomized controlled trial
Source: PLoS One. 2023 Jul 27;18(7):e0288436. doi: 10.1371/journal.pone.0288436 (PMC10373992; doi:10.1371/journal.pone.0288436)
Supplement: S2 Appendix — (DOCX) [file pone.0288436.s002.docx]

**APPENDIX B.1**

**(Baseline/Pre-Intervention Session – English Version)**

**DEMOGRAPHICS**

**1. Date of Birth:** / / 1 9 (Month/Day/Year)

The following questions are about your gender identity, the sex you were assigned at birth, and your sexual orientation. We ask these questions to better understand the health and health care needs of people with different gender identities and sexual orientations.

**2. What is your current gender identity?**

□ Woman □ Man

□ Trans Woman (Male-to-Female) □ Trans Man (Female-to-Male)

□ Gender Nonbinary/Genderqueer □ Additional gender category

□ Don’t know/Not sure (please specify): ____________

□ Prefer not to say

**3. What sex were you assigned at birth on your original birth certificate?**

□ Female □ Male □ Intersex

□ Don’t know/Not sure □ Prefer not to say □ Other: ________________

**4. Do you think of yourself as:**

□ Straight or heterosexual □ Lesbian □ Gay □ Bisexual

□ None of these describe me  □ Other: ______________________

□ Don’t know / Not sure □ Prefer not to say

The National Institutes of Health (NIH), which funds this study, requires that we collect information about the Race and Ethnicity of study participants to ensure that our research is inclusive of a diverse population.

**5. Which of the following best describes your ethnicity? (check only one)**

□ Hispanic or Latino □ Not Hispanic or Latino □ Prefer not to say

**6. Which of the following best describe(s) your Race? (check one or more)**

□ White

□ Black or African American

□ Asian

□ American Indian/Native American/Alaska Native □ Other: ______________

□ Hawaiian/Pacific Islander □ Unknown

□ More than one race □ Prefer not to say

**7. Do you consider English your first language? (check one)**

□ Yes □ No □ Prefer not to say

**7a. If English is NOT your first language, how well do you speak English?**

□ Not at all

□ Poorly

□ Fairly well

□ Well

□ Very well

□ N/A English is my first language

**7b. If English is NOT your first language, what is your first language? ___________**

**8. What is your household income?**

- $0
- $1 to $9,999
- $10,000 to $24,999
- $25,000 to $49,999
- $50,000 to $74,999
- $75,000 to $99,999
- $100,000 to $149,999
- $150,000 or more
- Unknown
- Prefer not to say

**9. Which of the following best describes your living situation?**

- Live in a house/apartment alone
- Live in a house/apartment with spouse and/or family
- Live in a house/apartment with a roommate(s)
- Live in a nursing home or assisted living facility
- Temporary/transitional housing
- Other (please specify): _________________________________
- Prefer not to say

**10. What is your partner/marital status? (check one)**

□ Single

□ Married (Partner / Spouse)

□ Separated

□ Widowed

□ Divorced

□ Never Married

□ Member of an unmarried couple

□ Prefer not to say

**10a. Are you currently living with your spouse or partner (if applicable)?**

□ Yes

□ No

□ Prefer not to say

**10b. What is your relationship to your loved one who experienced a cardiac arrest? (Check one)**

□ Partner / Spouse

□ Child

□ Stepchild

□ Sibling

□ Stepsibling

□ Parent

□ Stepparent

□ Aunt / Uncle

□ Grandparent

□ Godparent

□ Other ______________

□ Prefer not to say

**10c. Do you live with your loved one?**

□ Yes

□ No

□ Prefer not to say

**10d. Were you with your loved one during the following (check all that apply):**

□ Witnessed the event as it happened

□ Found loved one unresponsive

□ Witnessed or participated in resurrection efforts

□ Other

□ None of the above

□ Prefer not to say

**11. How long is your current relationship with your partner (if applicable)? ________ years**

**11a. How satisfied are you with your romantic relationship (if applicable)?**

**** Not at all  A little bit  Moderately  Extremely

**11b. How satisfied are you with your partner/spouse (if applicable)?**

**** Not at all  A little bit  Moderately  Extremely

**11c. How satisfied are you with your romantic relationship with your partner/spouse (if applicable)?**

**** Not at all  A little bit  Moderately  Extremely

**12. What is the highest grade or year of school you have completed?**

□ Less than high school

□ Some high school

□ High school diploma/GED

□ Trade school/Vocational school

□ Some college

□ College graduate

□ Graduate school/professional school

□ Associate’s Degree

□ No formal education

□ Prefer not to say

**13. Do you have health insurance?**

- Yes
- No
- Unsure
- Prefer not to say

**13a. In the past two years, have you always had health insurance or other coverage for medical care?**

□ Yes □No □Not Sure □Prefer not to say

**If NO:**

**13b. For how much time during the past two years did you not have coverage?**

□ Less than 6 months

□ 6 months - 1 year

□ More than 1 year

□ N/A Always had health insurance

**13c. Was there any time during the past two years when you DID NOT seek medical care because it was too expensive, or health insurance did not cover it?** Please do not include dental care.

□ Yes □No □Not Sure □Prefer not to say

**14. What is your home zip code? _____________**

**15. What is/was your occupation on your main job? (For example: registered nurse, personnel manager, auto mechanic, accountant, machine operator, etc.)**

**Occupation: ____________________________________**

**16. Please rate your agreement with the following statement.**

**I consider myself to be a religious and/or spiritual person.**

□ Strongly Disagree □ Disagree □ Neither agree nor disagree

□ Agree □ Strongly agree □ Prefer not to say

The following questions ask about how you were feeling during the cardiac event that brought your loved one to the hospital. **Please indicate to what extent you agree with the following statements.** As people are very different, there are no correct answers.

|  | **Not at all** | **A little bit** | **Moderately** | **Extremely** |
| --- | --- | --- | --- | --- |
| **I feel vulnerable.** | ➀ | ➁ | ➂ | ➃ |
| **I am worried that I am not in control.** | ➀ | ➁ | ➂ | ➃ |
| **I am worried that my loved one’s symptoms are severe.** | ➀ | ➁ | ➂ | ➃ |
| **I feel helpless.** | ➀ | ➁ | ➂ | ➃ |
| **I am worried that my loved one is going to die.** | ➀ | ➁ | ➂ | ➃ |
| **I am afraid.** | ➀ | ➁ | ➂ | ➃ |
| **I think this event will have a big impact on my life.** | ➀ | ➁ | ➂ | ➃ |

**APPENDIX B.2**

**(Baseline/Pre-Intervention Session – Spanish Version)**

**DATOS DEMOGRÁFICOS**

**1. Fecha de nacimiento:** / / 1 9 (Mes/Día/Año)

Las siguientes preguntas tratan sobre su identidad de género, el sexo que le fue asignado al momento de nacer, y su orientación sexual. Hacemos estas preguntas para poder entender mejor la salud y las necesidades de cuidado de las personas con diferentes identidades de género y orientaciones sexuales.

**2. ¿Cuál es su identidad de género?**

□ Mujer □ Hombre

□ Mujer transgénero (Hombre a Mujer) □ Hombre transgénero (Mujer a Hombre)

□ Género no binario / Genderqueer □ Otra identidad de género (especifique): ________

□ No lo sé/No estoy seguro/a □ Prefiero no decirlo

**3. ¿Cuál es el sexo que le asignaron en el momento de nacer en su certificado de nacimiento original?**

□ Hembra □ Varón □ Intersexual_________

□ No lo sé/No estoy seguro/a □ Prefiero no decirlo □ Otro: ________________

**4. Usted se considera:**

□ Heterosexual (*straight*) □ Lesbiana □ Hombre homosexual/Gay □ Bisexual

□ Ninguno de estos me describe □ Otro/a: ______________________

□ No lo sé/No estoy seguro/a □ Prefiero no decirlo

Los Institutos Nacionales de la Salud (*The National Institutes of Health, NIH*), los cuales financian este estudio, requieren que recopilemos información sobre la raza y etnia de los participantes del estudio para asegurarse de que nuestro estudio incluya una población diversa.

**5. ¿Cuál de las siguientes describe mejor su etnia? (marque una)**

□ Hispano/a o Latino/a □ No Hispano/a o Latino/a □ Prefiero no decirlo

**6. ¿Cuál(es) de los siguientes describe(n) mejor su raza? (seleccione una o más)**

□ Blanco

□ Negro o Afroamericano

□ Asiático

□ Indio Americano/Nativo Americano/Nativo de Alaska □ Otro/a: _______________

□ Hawaiano/ Isleño del Pacifico □ Desconocido

□ Más de una raza □ Prefiero no decirlo

**7. ¿Considera usted el inglés su idioma natal? (marque una)**

□ Si □ No □ Prefiero no decirlo

**7a. Si inglés NO es su idioma natal, ¿qué tan bien habla inglés?**

□ Nada en absoluto □ Bien

□ Mal □ Muy Bien

□ Regular □ No corresponde, inglés es mi idioma natal

**7b. Si inglés NO es su idioma natal, ¿cuál es su idioma natal?**

**8. ¿Cuál es el ingreso de su hogar?**

 $0

 $1 a $9,999

 $10,000 a $24,999

 $25,000 a $49,999

 $50,000 a $74,999

 $75,000 a $99,999

 $100,000 a $149,999

 $150,000 o más

 Desconocido

 Prefiero no decir

**9. ¿Cuál de las siguientes describe mejor su situación de vida?**

 Vive solo en una casa/apartamento

 Vive en una casa/apartamento con su cónyuge y/o familia

 Vive en una casa/apartamento con un compañero(s) de cuarto

 Vivir en un hogar de ancianos o centro de vida asistida

 Vivienda temporal/de transición

 Otro (por favor especifique): _________________________________

 Prefiero no decir

**10. ¿Cuál es su estado de pareja/estado civil? (marque uno)**

□ Soltero/a

□ Casado/a /emparejado/a

□ Separado/a

□ Viudo/a

□ Divorciado/a

□ Nunca casado/a

□ Miembro de una pareja no casada

□ Prefiero no decirlo

**10a. ¿Vive actualmente con su esposo/a o con su pareja (si aplica)?**

□ Sí

□ No

□ Prefiero no decirlo

**10b. ¿Cuál es su relación con su ser querido que sufrió un paro cardíaco? (marque uno)**

□ Pareja / Cónyuge

□ Niño

□ Hijastro

□ Hermano

□ Hermanastro

□ Padre

□ Padrastro

□ Tía / Tío

□ Abuelo

□ Padrino

□ Otro ______________

□ Prefiero no decir

**10c.** **¿Vive con su ser querido?**

□ Sí

□ No

□ Prefiero no decir

**10d. Estuvo con su ser querido durante lo siguiente (marque todo lo que corresponda):**

□ Estuvo presente cuando el evento sucedió

□ Encontró a su ser querido inconsciente

□ Fue testigo de o participó en los esfuerzos de resurrección/reanimación

□ Otro

□ Ninguna de las anteriores

□ Prefiero no decirlo

**11. ¿Cuántos años lleva en su relación con su pareja actual (si aplica)? ____________ años**

**11a. ¿Qué tan satisfecho/a está usted con su relación romántica (si aplica)?**

**** Nada en absoluto  Un poco  Moderadamente  Extremadamente

**11b. ¿Qué tan satisfecho/a está usted con su pareja/esposo/a (si aplica)?**

**** Nada en absoluto  Un poco  Moderadamente  Extremadamente

**11c. ¿Qué tan satisfecho/a está usted con su relación romántica con su pareja/esposo/a (si aplica)?**

**** Nada en absoluto  Un poco  Moderadamente  Extremadamente

**12. ¿Cuál es el grado (o año) más alto de escuela que usted ha completado? (marque uno)**

□ Menos de escuela secundaria

□ Algunos años de escuela secundaria

□ Escuela secundaria/GED

□ Escuela comercial o escuela vocacional

□ Algunos años de universidad

□ Graduado universitario

□ Graduado de escuela posgraduada o escuela profesional

□ Graduado de asociado

□ Ninguna educación formal

□ Prefiero no decirlo

**13. ¿Tiene seguro médico?**

**** Sí

 No

 No estoy seguro

 Prefiero no decir

**13a. Durante los últimos 2 años, ¿ha tenido siempre un seguro de salud u otra cobertura de atención médica?**

□ Sí □ No □ No lo sé/No estoy seguro/a □ Prefiero no decirlo

**Si respondió NO:**

**13b. ¿Por cuánto tiempo durante los últimos 2 años no tuvo cobertura?**

□ Menos de 6 meses □ 6 meses - 1 año

□ Más de 1 año □ No corresponde, siempre he tenido seguro médico

**13c. ¿Hubo algún tiempo durante los últimos 2 años durante el cual NO buscó atención médica porque era demasiado caro o el seguro de salud no lo cubría?** Por favor, no incluya el cuidado dental.

□ Sí □No □ No lo sé/No estoy seguro/a □ Prefiero no decirlo

**14. ¿Cuál es el código postal de su casa? _____________**

**15. ¿Cuál es/era su ocupación en su trabajo principal? (Por ejemplo: enfermera registrada, gerente de personal, mecánico de automóviles, contador, operador de máquinas, etc.)**

**Ocupación:**

**16.** **Por favor, valore su acuerdo con la siguiente afirmación.**

**Me considero una persona religiosa y/o espiritual.**

**** Muy en desacuerdo  En desacuerdo  Ni de acuerdo ni en desacuerdo

 De acuerdo  Muy de acuerdo  Prefiero no decirlo

Las siguientes preguntas se refieren a cómo se sintió usted durante el evento cardíaco que llevó a su ser querido al hospital. **Por favor, indique en qué medida está de acuerdo con las siguientes afirmaciones.** Como las personas son muy diferentes, no hay respuestas correctas.

|  | **Nada en absoluto** | **Un poco** | **Moderadamente** | **Extremadamente** |
| --- | --- | --- | --- | --- |
| **Me siento vulnerable (indefenso).** | ➀ | ➁ | ➂ | ➃ |
| **Me preocupa que no tengo el control.** | ➀ | ➁ | ➂ | ➃ |
| **Me preocupa que los síntomas de mi ser querido sean graves.** | ➀ | ➁ | ➂ | ➃ |
| **Me siento desamparado/a (desvalido/a).** | ➀ | ➁ | ➂ | ➃ |
| **Me preocupa que mi ser querido se vaya a morir.** | ➀ | ➁ | ➂ | ➃ |
| **Tengo miedo.** | ➀ | ➁ | ➂ | ➃ |
| **Creo que este evento tendrá un gran impacto en mi vida.** | ➀ | ➁ | ➂ | ➃ |

**APPENDIX B.3**

**(Post-Intervention Session – English Version)**

**Date of patient discharge from hospital: _____/_____/_____**

**Patient disposition: ____** Living **____** Deceased

**PHQ-2 and GAD-2**

**Instructions:** Over the last week, how often have you been bothered by the following problems?

|  | **Not at all** | **Several days** | **More than half the days** | **Nearly every day** |
| --- | --- | --- | --- | --- |
| 1. Little interest or pleasure in doing things. | ➀ | ➁ | ➂ | ➃ |
| 2. Feeling down, depressed or hopeless. | ➀ | ➁ | ➂ | ➃ |
| 3. Feeling nervous, anxious or on edge. | ➀ | ➁ | ➂ | ➃ |
| 4. Not being able to stop or control worrying. | ➀ | ➁ | ➂ | ➃ |

**Fear** *(only ask if patient is surviving)*

**INSTRUCTIONS:** The items below ask about your thoughts and feelings about your loved one following their cardiac event. Please rank each statement below by selecting one option from 1 to 5 based on how much it is true for you. A ranking of 5 means this *always* is true for you, and 1 means it *never* is true for you. Be sure to consider the entire range of the scale (1, 2, 3, 4, or 5) when you respond to each statement.

|  | **Never** | **Rarely** | **Sometimes** | **Often** | **Always** |
| --- | --- | --- | --- | --- | --- |
| 1. If my loved one’s tests come out normal, I still worry about their heart. | ➀ | ➁ | ➂ | ➃ | ➄ |
| 2. I feel safe when my loved one is around a hospital, physician or other medical facility. | ➀ | ➁ | ➂ | ➃ | ➄ |
| 3. I worry that doctors do not believe my loved one’s chest pain/discomfort is real. | ➀ | ➁ | ➂ | ➃ | ➄ |
| **When my loved one has chest discomfort, or when their heart is beating fast:** |  |  |  |  |  |
| 4. … I worry that my loved one may have a heart attack. | ➀ | ➁ | ➂ | ➃ | ➄ |
| 5. … I have difficulty concentrating on anything else. | ➀ | ➁ | ➂ | ➃ | ➄ |
| 6. … I get frightened. | ➀ | ➁ | ➂ | ➃ | ➄ |
| 7. … I like my loved one to be checked out by a doctor. | ➀ | ➁ | ➂ | ➃ | ➄ |
| 8. … I tell my family or friends. | ➀ | ➁ | ➂ | ➃ | ➄ |

**Avoidance** *(only ask if patient is surviving)*

**INSTRUCTIONS:** The items below ask about your thoughts and feelings about your loved one following their cardiac event. Please rank each statement below by selecting one option from 1 to 5 based on how much it is true for you. A ranking of 5 means this *always* is true for you, and 1 means it *never* is true for you. Be sure to consider the entire range of the scale (1, 2, 3, 4, or 5) when you respond to each statement.

|  | **Never** | **Rarely** | **Sometimes** | **Often** | **Always** |
| --- | --- | --- | --- | --- | --- |
| 1. My loved one should avoid physical exertion. | ➀ | ➁ | ➂ | ➃ | ➄ |
| 2. My loved one should take it easy as much as possible. | ➀ | ➁ | ➂ | ➃ | ➄ |
| 3. My loved one should avoid exercise or other physical work. | ➀ | ➁ | ➂ | ➃ | ➄ |
| 4. My loved one should avoid activities that make their heart beat faster. | ➀ | ➁ | ➂ | ➃ | ➄ |
| 5. My loved one should avoid activities that make them sweat. | ➀ | ➁ | ➂ | ➃ | ➄ |

**APPENDIX B.4**

**(Post-Intervention Session – Spanish Version)**

**Date of patient discharge from hospital: _____/_____/_____**

**Patient disposition: ____** Living **____** Deceased

**PHQ-2 and GAD-2**

**Instrucciones:** Durante la última semana, ¿con qué frecuencia le han molestado los siguientes problemas?

|  | **Ningún Día** | **Varios Días** | **Más de la mitad de los días** | **Casi todos los días** |
| --- | --- | --- | --- | --- |
| 1. Tener poco interés o placer en hacer cosas. | ➀ | ➁ | ➂ | ➃ |
| 2. Sentirse desanimado(a), deprimido(a) o sin esperanza. | ➀ | ➁ | ➂ | ➃ |
| 3. Sentirse nervioso(a), con ansiedad o con los nervios de punta (sentirse en el límite). | ➀ | ➁ | ➂ | ➃ |
| 4. No ser capaz de parar de preocuparse o de controlar sus preocupaciones. | ➀ | ➁ | ➂ | ➃ |

**Miedo** *(only ask if patient is surviving)*

**INSTRUCCIONES:** Las siguientes frases le preguntan sobre sus pensamientos y sentimientos acerca de su ser querido después del evento cardíaco. Por favor clasifique cada afirmación seleccionando una opción del 1 al 5 basándose en cuánto es cierto cada afirmación para usted. Una puntuación de 5 significa que esto *siempre* es cierto para usted, y una puntuación de 1 significa que *nunca* es cierto para usted. Asegúrese de considerar todas las opciones (1, 2, 3, 4 o 5) cuando responda a cada afirmación.

|  | **Nunca** | **Casi Nunca** | **Algunas Veces** | **Con Frecuencia** | **Siempre** |
| --- | --- | --- | --- | --- | --- |
| 1. Si los resultados de los exámenes de mi ser querido resultan normales, todavía me preocupo por su corazón. | ➀ | ➁ | ➂ | ➃ | ➄ |
| 2. Me siento seguro/a cuando mi ser querido está cerca de un hospital, médico u otro centro médico. | ➀ | ➁ | ➂ | ➃ | ➄ |
| 3. Me preocupa que los médicos no crean que el dolor/malestar en el pecho de mi ser querido sea real. | ➀ | ➁ | ➂ | ➃ | ➄ |
| **Cuando mi ser querido tiene molestia/ malestar en el pecho o cuando su corazón late rápido:** |  |  |  |  |  |
| 4. …me preocupa que mi ser querido pueda sufrir un ataque al corazón. | ➀ | ➁ | ➂ | ➃ | ➄ |
| 5. …tengo dificultad para concentrarme en cualquier otra cosa. | ➀ | ➁ | ➂ | ➃ | ➄ |
| 6. … me asusto. | ➀ | ➁ | ➂ | ➃ | ➄ |
| 7. …me gusta que mi ser querido sea examinado por un médico. | ➀ | ➁ | ➂ | ➃ | ➄ |
| 8. …le cuento a mi familia o amigos/as. | ➀ | ➁ | ➂ | ➃ | ➄ |

**Evitación** *(only ask if patient is surviving)*

**INSTRUCCIONES:** Las siguientes frases le preguntan sobre sus pensamientos y sentimientos acerca de su ser querido después del evento cardíaco. Por favor clasifique cada afirmación seleccionando una opción del 1 al 5 basándose en cuánto es cierto cada afirmación para usted. Una puntuación de 5 significa que esto *siempre* es cierto para usted, y una puntuación de 1 significa que *nunca* es cierto para usted. Asegúrese de considerar todas las opciones (1, 2, 3, 4 o 5) cuando responda a cada afirmación.

|  | **Nunca** | **Casi Nunca** | **Algunas Veces** | **Con Frecuencia** | **Siempre** |
| --- | --- | --- | --- | --- | --- |
| 1. Mi ser querido debería evitar el esfuerzo físico. | ➀ | ➁ | ➂ | ➃ | ➄ |
| 2. Mi ser querido debería tomarlo con calma tanto como sea posible. | ➀ | ➁ | ➂ | ➃ | ➄ |
| 3. Mi ser querido debería evitar el ejercicio u otro trabajo físico. | ➀ | ➁ | ➂ | ➃ | ➄ |
| 4. Mi ser querido debería evitar las actividades que hacen que su corazón palpite más rápido. | ➀ | ➁ | ➂ | ➃ | ➄ |
| 5. Mi ser querido debería evitar actividades que le hagan sudar. | ➀ | ➁ | ➂ | ➃ | ➄ |

**APPENDIX B.5**

**(Follow-Up Session – English Version)**

**PHQ-2 and GAD-2**

**Instructions:** Over the last week, how often have you been bothered by the following problems?

|  | **Not at all** | **Several days** | **More than half the days** | **Nearly every day** |
| --- | --- | --- | --- | --- |
| 1. Little interest or pleasure in doing things. | ➀ | ➁ | ➂ | ➃ |
| 2. Feeling down, depressed or hopeless. | ➀ | ➁ | ➂ | ➃ |
| 3. Feeling nervous, anxious or on edge. | ➀ | ➁ | ➂ | ➃ |
| 4. Not being able to stop or control worrying. | ➀ | ➁ | ➂ | ➃ |

**Fear** *(only ask if patient is surviving)*

**INSTRUCTIONS:** The items below ask about your thoughts and feelings about your loved one following their cardiac event. Please rank each statement below by selecting one option from 1 to 5 based on how much it is true for you. A ranking of 5 means this *always* is true for you, and 1 means it *never* is true for you. Be sure to consider the entire range of the scale (1, 2, 3, 4, or 5) when you respond to each statement.

|  | **Never** | **Rarely** | **Sometimes** | **Often** | **Always** |
| --- | --- | --- | --- | --- | --- |
| 1. If my loved one’s tests come out normal, I still worry about their heart. | ➀ | ➁ | ➂ | ➃ | ➄ |
| 2. I feel safe when my loved one is around a hospital, physician or other medical facility. | ➀ | ➁ | ➂ | ➃ | ➄ |
| 3. I worry that doctors do not believe my loved one’s chest pain/discomfort is real. | ➀ | ➁ | ➂ | ➃ | ➄ |
| **When my loved one has chest discomfort, or when their heart is beating fast:** |  |  |  |  |  |
| 4. … I worry that my loved one may have a heart attack. | ➀ | ➁ | ➂ | ➃ | ➄ |
| 5. … I have difficulty concentrating on anything else. | ➀ | ➁ | ➂ | ➃ | ➄ |
| 6. … I get frightened. | ➀ | ➁ | ➂ | ➃ | ➄ |
| 7. … I like my loved one to be checked out by a doctor. | ➀ | ➁ | ➂ | ➃ | ➄ |
| 8. … I tell my family or friends. | ➀ | ➁ | ➂ | ➃ | ➄ |

**Avoidance** *(only ask if patient is surviving)*

**INSTRUCTIONS:** The items below ask about your thoughts and feelings about your loved one following their cardiac event. Please rank each statement below by selecting one option from 1 to 5 based on how much it is true for you. A ranking of 5 means this *always* is true for you, and 1 means it *never* is true for you. Be sure to consider the entire range of the scale (1, 2, 3, 4, or 5) when you respond to each statement.

|  | **Never** | **Rarely** | **Sometimes** | **Often** | **Always** |
| --- | --- | --- | --- | --- | --- |
| 1. My loved one should avoid physical exertion. | ➀ | ➁ | ➂ | ➃ | ➄ |
| 2. My loved one should take it easy as much as possible. | ➀ | ➁ | ➂ | ➃ | ➄ |
| 3. My loved one should avoid exercise or other physical work. | ➀ | ➁ | ➂ | ➃ | ➄ |
| 4. My loved one should avoid activities that make their heart beat faster. | ➀ | ➁ | ➂ | ➃ | ➄ |
| 5. My loved one should avoid activities that make them sweat. | ➀ | ➁ | ➂ | ➃ | ➄ |

**PTSS**

Many people feel distressed after experiencing a loved one having a cardiac arrest. Which of the following was the most distressing part of your loved one’s cardiac arrest experience?

 The heart problem/symptoms your loved one experienced that brought you to the hospital

 The experience in the Emergency Department

 The experience in the ICU

 The experience after the ICU

**INSTRUCTIONS:** Below is a list of problems and complaints that people sometimes have in response to stressful life experiences. Please read/listen to each one carefully and indicate how much you have been bothered by that problem in the LAST MONTH in response to **the distressing cardiac arrest experience you indicated above**. Please answer using the following options: (1) Not at all; (2) A little bit; (3) Moderately; (4) Quite a bit; (5) Extremely

| **In the past month, how much were you bothered by…** | **Not at all** | **A little bit** | **Moderately** | **Quite a bit** | **Extremely** |
| --- | --- | --- | --- | --- | --- |
| 1. Repeated, disturbing, and unwanted memories of the stressful experience? | ➀ | ➁ | ➂ | ➃ | ➄ |
| 2. Repeated, disturbing dreams of the stressful experience? | ➀ | ➁ | ➂ | ➃ | ➄ |
| 3. Suddenly acting or feeling as if the stressful experience were actually happening again (as if you were actually back there reliving it)? | ➀ | ➁ | ➂ | ➃ | ➄ |
| 4. Feeling very upset when something reminded you of the stressful experience? | ➀ | ➁ | ➂ | ➃ | ➄ |
| 5. Having physical reactions when something reminded you of the stressful experience (e.g., heart pounding, trouble breathing, sweating)? | ➀ | ➁ | ➂ | ➃ | ➄ |
| 6. Avoiding memories, thoughts, or feelings related to the stressful experience? | ➀ | ➁ | ➂ | ➃ | ➄ |
| 7. Avoiding external reminders of the stressful experience (e.g., people, places, conversations, activities, objects, or situations)? | ➀ | ➁ | ➂ | ➃ | ➄ |
| 8. Trouble remembering important parts of the stressful experience? | ➀ | ➁ | ➂ | ➃ | ➄ |

| **In the past month, how much were you bothered by…** | **Not at all** | **A little bit** | **Moderately** | **Quite a bit** | **Extremely** |
| --- | --- | --- | --- | --- | --- |
| 9. Having strong negative beliefs about yourself, other people, or the world (e.g., having thoughts such as: I am bad, there is something seriously wrong with me, no one can be trusted, the world is completely dangerous)? | ➀ | ➁ | ➂ | ➃ | ➄ |
| 10. Blaming yourself or someone else strongly for the stressful experience or what happened after it? | ➀ | ➁ | ➂ | ➃ | ➄ |
| 11. Having strong negative feelings such as fear, horror, anger, guilt, or shame? | ➀ | ➁ | ➂ | ➃ | ➄ |
| 12. Loss of interest in activities that you used to enjoy? | ➀ | ➁ | ➂ | ➃ | ➄ |
| 13. Feeling distant or cut off from other people? | ➀ | ➁ | ➂ | ➃ | ➄ |
| 14. Having trouble experiencing positive feelings (e.g., being unable to have loving feelings for those close to you, or feeling emotionally numb)? | ➀ | ➁ | ➂ | ➃ | ➄ |
| 15. Feeling irritable or angry or acting aggressively? | ➀ | ➁ | ➂ | ➃ | ➄ |
| 16. Taking too many risks or doing things that cause you harm? | ➀ | ➁ | ➂ | ➃ | ➄ |
| 17. Being “super alert” or watchful or on guard? | ➀ | ➁ | ➂ | ➃ | ➄ |
| 18. Feeling jumpy or easily startled? | ➀ | ➁ | ➂ | ➃ | ➄ |
| 19. Having difficulty concentrating? | ➀ | ➁ | ➂ | ➃ | ➄ |
| 20. Trouble falling or staying asleep? | ➀ | ➁ | ➂ | ➃ | ➄ |

**Appropriateness, Feasibility, and Acceptability (intervention participants only)**

**INSTRUCTIONS:** For each statement below, **“the ICU diary”** refers to the **diary you completed while your loved one was in the ICU**. Please rank each statement below by selecting one option from 1 to 5 based on how much you agree with it. A ranking of 5 means you *completely agree*, and 1 means you *completely disagree*. Be sure to consider the entire range of the scale (1, 2, 3, 4, or 5) when you respond to each statement.

|  | **Completely disagree** | **Disagree** | **Neither agree nor disagree** | **Agree** | **Completely agree** |
| --- | --- | --- | --- | --- | --- |
| 1. The ICU diary seems fitting for reducing fear about my loved one’s heart. | ➀ | ➁ | ➂ | ➃ | ➄ |
| 2. The ICU diary seems suitable for reducing fear about my loved one’s heart. | ➀ | ➁ | ➂ | ➃ | ➄ |
| 3. The ICU diary seems applicable for reducing fear about my loved one’s heart. | ➀ | ➁ | ➂ | ➃ | ➄ |
| 4. The ICU diary seems like a good match for reducing fear about my loved one’s heart. | ➀ | ➁ | ➂ | ➃ | ➄ |

|  | **Completely disagree** | **Disagree** | **Neither agree nor disagree** | **Agree** | **Completely agree** |
| --- | --- | --- | --- | --- | --- |
| 5. The ICU diary seems implementable. | ➀ | ➁ | ➂ | ➃ | ➄ |
| 6. The ICU diary seems possible. | ➀ | ➁ | ➂ | ➃ | ➄ |
| 7. The ICU diary seems doable. | ➀ | ➁ | ➂ | ➃ | ➄ |
| 8. The ICU diary seems easy to use. | ➀ | ➁ | ➂ | ➃ | ➄ |

|  | **Completely disagree** | **Disagree** | **Neither agree nor disagree** | **Agree** | **Completely agree** |
| --- | --- | --- | --- | --- | --- |
| 9. The ICU diary meets my approval. | ➀ | ➁ | ➂ | ➃ | ➄ |
| 10. The ICU diary is appealing to me. | ➀ | ➁ | ➂ | ➃ | ➄ |
| 11. I like the ICU diary. | ➀ | ➁ | ➂ | ➃ | ➄ |
| 12. I welcome the ICU diary. | ➀ | ➁ | ➂ | ➃ | ➄ |

|  | **Completely disagree** | **Disagree** | **Neither agree nor disagree** | **Agree** | **Completely agree** |
| --- | --- | --- | --- | --- | --- |
| 13. It was easy to think of topics to write about in the ICU diary. | ➀ | ➁ | ➂ | ➃ | ➄ |
| 14. It was easy to find time to write in the ICU diary. | ➀ | ➁ | ➂ | ➃ | ➄ |
| 15. Writing in the ICU diary made me feel better. | ➀ | ➁ | ➂ | ➃ | ➄ |

IF A CONTROL PARTICIPANT: Did you keep a diary while your loved one was in the ICU? ____ Yes ____ No

*If yes, ask the following questions. Otherwise only ask the questions of the intervention group.*

How often did you write in the diary? ____ times/week

Was this the same during the ICU stay? ____ times/week

After discharge? ____ times/week

IF THE PATIENT IS LIVING: Did you share the ICU diary with your loved one?

___ Yes ___ No

If **YES**: Is there anything you would like to share about your experience sharing the ICU diary with your loved one? ________________________________________________________________________________________________________________________________________________

If **NO**: Do you plan to share the ICU diary with the patient? ____ Yes ____ No

Is there anything you liked about the ICU diary that you would like to share? ____________________________________________________________________________________________________________________________________________________________

Were there any challenges you would like to share? ____________________________________________________________________________________________________________________________________________________________

If you were to give someone advice for writing an ICU diary, what would you tell them? ____________________________________________________________________________________________________________________________________________________________

If you could change anything about the ICU diary, what would you change? ____________________________________________________________________________________________________________________________________________________________

Is there anything else you would like to share about your experience writing in the diary? ____________________________________________________________________________________________________________________________________________________________

**Please rate your agreement with the following statements.**

**1. My religious and/or spiritual beliefs have helped me cope with my loved one’s cardiac event.**

□ Strongly Disagree □ Disagree □ Neither agree nor disagree

□ Agree □ Strongly agree □ Prefer not to say

**2. My loved one’s cardiac event has weakened my religious and/or spiritual beliefs.**

□ Strongly Disagree □ Disagree □ Neither agree nor disagree

□ Agree □ Strongly agree □ Prefer not to say

**3. My loved one’s cardiac event has strengthened my religious and/or spiritual beliefs.**

□ Strongly Disagree □ Disagree □ Neither agree nor disagree

□ Agree □ Strongly agree □ Prefer not to say

**FUTURE CONTACT**

1. Are you willing to be contacted by our Center about future research studies? (Y/N)

(If yes to #1): Are you willing to be contacted by our Center in the future to provide feedback about the design of research studies from a participant’s perspective?  (Y/N)

**APPENDIX B.6**

**(Follow-Up Session – Spanish Version)**

**PHQ-2 and GAD-2**

**Instrucciones:** Durante la última semana, ¿con qué frecuencia le han molestado los siguientes problemas?

|  | **Ningún Día** | **Varios Días** | **Más de la mitad de los días** | **Casi todos los días** |
| --- | --- | --- | --- | --- |
| 1. Tener poco interés o placer en hacer cosas. | ➀ | ➁ | ➂ | ➃ |
| 2. Sentirse desanimado(a), deprimido(a) o sin esperanza. | ➀ | ➁ | ➂ | ➃ |
| 3. Sentirse nervioso(a), con ansiedad o con los nervios de punta (sentirse en el límite). | ➀ | ➁ | ➂ | ➃ |
| 4. No ser capaz de parar de preocuparse o de controlar sus preocupaciones. | ➀ | ➁ | ➂ | ➃ |

**Miedo** *(only ask if patient is surviving)*

**INSTRUCCIONES:** Las siguientes frases le preguntan sobre sus pensamientos y sentimientos acerca de su ser querido después del evento cardíaco. Por favor clasifique cada afirmación seleccionando una opción del 1 al 5 basándose en cuánto es cierto cada afirmación para usted. Una puntuación de 5 significa que esto *siempre* es cierto para usted, y una puntuación de 1 significa que *nunca* es cierto para usted. Asegúrese de considerar todas las opciones (1, 2, 3, 4 o 5) cuando responda a cada afirmación.

|  | **Nunca** | **Casi Nunca** | **Algunas Veces** | **Con Frecuencia** | **Siempre** |
| --- | --- | --- | --- | --- | --- |
| 1. Si los resultados de los exámenes de mi ser querido resultan normales, todavía me preocupo por su corazón. | ➀ | ➁ | ➂ | ➃ | ➄ |
| 2. Me siento seguro/a cuando mi ser querido está cerca de un hospital, médico u otro centro médico. | ➀ | ➁ | ➂ | ➃ | ➄ |
| 3. Me preocupa que los médicos no crean que el dolor/malestar en el pecho de mi ser querido sea real. | ➀ | ➁ | ➂ | ➃ | ➄ |
| **Cuando mi ser querido tiene molestia/ malestar en el pecho o cuando su corazón late rápido:** |  |  |  |  |  |
| 4. …me preocupa que mi ser querido pueda sufrir un ataque al corazón. | ➀ | ➁ | ➂ | ➃ | ➄ |
| 5. …tengo dificultad para concentrarme en cualquier otra cosa. | ➀ | ➁ | ➂ | ➃ | ➄ |
| 6. … me asusto. | ➀ | ➁ | ➂ | ➃ | ➄ |
| 7. …me gusta que mi ser querido sea examinado por un médico. | ➀ | ➁ | ➂ | ➃ | ➄ |
| 8. …le cuento a mi familia o amigos/as. | ➀ | ➁ | ➂ | ➃ | ➄ |

**Evitación** *(only ask if patient is surviving)*

**INSTRUCCIONES:** Las siguientes frases le preguntan sobre sus pensamientos y sentimientos acerca de su ser querido después del evento cardíaco. Por favor clasifique cada afirmación seleccionando una opción del 1 al 5 basándose en cuánto es cierto cada afirmación para usted. Una puntuación de 5 significa que esto *siempre* es cierto para usted, y una puntuación de 1 significa que *nunca* es cierto para usted. Asegúrese de considerar todas las opciones (1, 2, 3, 4 o 5) cuando responda a cada afirmación.

|  | **Nunca** | **Casi Nunca** | **Algunas Veces** | **Con Frecuencia** | **Siempre** |
| --- | --- | --- | --- | --- | --- |
| 1. Mi ser querido debería evitar el esfuerzo físico. | ➀ | ➁ | ➂ | ➃ | ➄ |
| 2. Mi ser querido debería tomarlo con calma tanto como sea posible. | ➀ | ➁ | ➂ | ➃ | ➄ |
| 3. Mi ser querido debería evitar el ejercicio u otro trabajo físico. | ➀ | ➁ | ➂ | ➃ | ➄ |
| 4. Mi ser querido debería evitar las actividades que hacen que su corazón palpite más rápido. | ➀ | ➁ | ➂ | ➃ | ➄ |
| 5. Mi ser querido debería evitar actividades que le hagan sudar. | ➀ | ➁ | ➂ | ➃ | ➄ |

**Síndrome de Estrés Postraumático**

Muchas personas se sienten angustiadas después de que su ser querido experimenta un paro cardíaco. ¿Cuál de las siguientes fue la parte más angustiosa de la experiencia del paro cardíaco de su ser querido?

 El problema del corazón o los síntomas del corazón que experimentó su ser querido que lo llevaron al hospital.

 La experiencia en el Departamento de Emergencias

 La experiencia en la Unidad de Cuidados Intensivos (UCI)

 La experiencia *después* de estar en la Unidad de Cuidados Intensivos (UCI)

**INSTRUCCIONES:** A continuación, se muestra una lista de problemas y quejas que las personas a veces tienen en respuesta a experiencias estresantes de la vida. Lea / escuche atentamente a cada uno e indique cuánto le ha molestado ese problema en el ÚLTIMO MES en respuesta a **la angustiosa experiencia del paro cardíaco que indicó anteriormente.** Responda utilizando las siguientes opciones: (1) Para nada; (2) Un poco; (3) Moderadamente; (4) Bastante; (5) Extremadamente

| **En el último mes, ¿cuánto le ha molestado:** | **Nada** | **Un Poco** | **Modera-damente** | **Bastante** | **Extrema-damente** |
| --- | --- | --- | --- | --- | --- |
| 1. … tener recuerdos repetidos, inquietantes/ alarmantes y no deseados sobre la experiencia estresante? | ➀ | ➁ | ➂ | ➃ | ➄ |
| 2. … tener sueños repetidos e inquietantes/ alarmantes sobre la experiencia estresante? | ➀ | ➁ | ➂ | ➃ | ➄ |
| 3. … sentir o actuar de repente como si la experiencia estresante ocurriera realmente otra vez (como si estuviera realmente ahí reviviéndola)? | ➀ | ➁ | ➂ | ➃ | ➄ |
| 4. … sentirse muy molesto(a) cuando algo le recuerda la experiencia estresante? | ➀ | ➁ | ➂ | ➃ | ➄ |
| 5. … tener fuertes reacciones físicas cuando algo le recuerda la experiencia estresante (por ejemplo, palpitaciones, dificultad para respirar, sudor)? | ➀ | ➁ | ➂ | ➃ | ➄ |
| 6. … evitar memorias, pensamientos o sentimientos relacionados con la experiencia estresante? | ➀ | ➁ | ➂ | ➃ | ➄ |
| 7. … evitar recordatorios externos de la experiencia estresante (por ejemplo, personas, lugares, conversaciones, actividades, objetos o situaciones)? | ➀ | ➁ | ➂ | ➃ | ➄ |
| 8. … tener problemas para recordar partes importantes de la experiencia estresante? | ➀ | ➁ | ➂ | ➃ | ➄ |

| **En el último mes, ¿cuánto le ha molestado:** | **Nada** | **Un Poco** | **Modera-damente** | **Bastante** | **Extrema-damente** |
| --- | --- | --- | --- | --- | --- |
| 9. … tener fuertes creencias negativas sobre sí mismo/a, otras personas o el mundo (por ejemplo, tener pensamientos como: soy malo/a, hay algo seriamente mal conmigo, no se puede confiar en nadie, el mundo es completamente peligroso)? | ➀ | ➁ | ➂ | ➃ | ➄ |
| 10. … culparse fuertemente a sí mismo/a o a otro por la experiencia estresante o por lo que ocurrió después? | ➀ | ➁ | ➂ | ➃ | ➄ |
| 11. … tener fuertes sentimientos negativos como miedo, horror, ira, culpa o vergüenza? | ➀ | ➁ | ➂ | ➃ | ➄ |
| 12. … perder el interés en actividades que antes disfrutaba? | ➀ | ➁ | ➂ | ➃ | ➄ |
| 13. … sentirse alejado/a o aislado/a de otras personas? |  |  |  |  |  |
| 14. … tener problemas para tener sentimientos positivos (por ejemplo, ser incapaz de tener sentimientos cariñosos hacia las personas cercanas a usted, o sentirse emocionalmente entumecido)? | ➀ | ➁ | ➂ | ➃ | ➄ |
| 15. … sentirse irritable o enojado/a o actuar agresivamente? | ➀ | ➁ | ➂ | ➃ | ➄ |
| 16. … arriesgarse demasiado o hacer cosas que le causan daño? | ➀ | ➁ | ➂ | ➃ | ➄ |
| 17. … sentirse super-alerta, vigilante o en guardia? | ➀ | ➁ | ➂ | ➃ | ➄ |
| 18. … sentirse nervioso/a o que se sobresalta fácilmente? | ➀ | ➁ | ➂ | ➃ | ➄ |
| 19. … tener problemas para concentrarse? | ➀ | ➁ | ➂ | ➃ | ➄ |
| 20. … tener problemas para dormir o seguir durmiendo? | ➀ | ➁ | ➂ | ➃ | ➄ |

**Adecuación, Viabilidad, y Aceptabilidad** *(intervention participants only)*

**INSTRUCCIONES:** En cada una de las afirmaciones siguientes, "**el diario de la UCI**" se refiere al **diario que usted completó mientras su ser querido estaba en la UCI**. Por favor, clasifique cada una de las afirmaciones que aparecen a continuación seleccionando una opción del 1 al 5 en función de cuánto esté de acuerdo con cada afirmación. Una puntuación de 5 significa que usted está *completamente de acuerdo*, y una puntuación de 1 significa que está *completamente en desacuerdo*. Asegúrese de considerar todo el rango de la escala (1, 2, 3, 4 o 5) cuando responda a cada afirmación.

|  | **Completamente en desacuerdo** | **En desacuerdo** | **Ni de acuerdo ni en desacuerdo** | **De acuerdo** | **Completamente de acuerdo** |
| --- | --- | --- | --- | --- | --- |
| 1. El diario de la UCI parece apropiado para reducir el miedo sobre el corazón de mi ser querido. | ➀ | ➁ | ➂ | ➃ | ➄ |
| 2. El diario de la UCI parece adecuado para reducir el miedo sobre el corazón de mi ser querido. | ➀ | ➁ | ➂ | ➃ | ➄ |
| 3. El diario de la UCI parece aplicable para reducir el miedo sobre el corazón de mi ser querido. | ➀ | ➁ | ➂ | ➃ | ➄ |
| 4. El diario de la UCI parece ser una buena combinación para reducir el miedo sobre el corazón de mi ser querido. | ➀ | ➁ | ➂ | ➃ | ➄ |

|  | **Completamente en desacuerdo** | **En**  **desacuerdo** | **Ni de acuerdo ni en desacuerdo** | **De acuerdo** | **Completamente de acuerdo** |
| --- | --- | --- | --- | --- | --- |
| 5. El diario de la UCI parece ser algo que se puede llevar a cabo (implementable). | ➀ | ➁ | ➂ | ➃ | ➄ |
| 6. El diario de la UCI parece ser algo posible. | ➀ | ➁ | ➂ | ➃ | ➄ |
| 7. El diario de la UCI parece que se puede hacer (factible/realizable/viable). | ➀ | ➁ | ➂ | ➃ | ➄ |
| 8. El diario de la UCI parece fácil de usar. | ➀ | ➁ | ➂ | ➃ | ➄ |

|  | **Completamente en desacuerdo** | **En desacuerdo** | **Ni de acuerdo ni en desacuerdo** | **De acuerdo** | **Completamente de acuerdo** |
| --- | --- | --- | --- | --- | --- |
| 9. El diario de la UCI tiene mi aprobación. | ➀ | ➁ | ➂ | ➃ | ➄ |
| 10. El diario de la UCI me atrae (me llama la atención). | ➀ | ➁ | ➂ | ➃ | ➄ |
| 11. Me gusta el diario de la UCI. | ➀ | ➁ | ➂ | ➃ | ➄ |
| 12. Le doy la bienvenida al diario de la UCI (“le abro la puerta”). | ➀ | ➁ | ➂ | ➃ | ➄ |

|  | **Completamente en desacuerdo** | **En desacuerdo** | **Ni de acuerdo ni en desacuerdo** | **De acuerdo** | **Completamente de acuerdo** |
| --- | --- | --- | --- | --- | --- |
| 13. Fue fácil pensar en temas para escribir en el diario de la UCI. | ➀ | ➁ | ➂ | ➃ | ➄ |
| 14. Fue fácil encontrar tiempo para escribir en el diario de la UCI. | ➀ | ➁ | ➂ | ➃ | ➄ |
| 15. Escribir en el diario de la UCI me hizo sentir mejor. | ➀ | ➁ | ➂ | ➃ | ➄ |

**IF A CONTROL PARTICIPANT:** ¿Escribió en un diario mientras su ser querido estaba en la Unidad de Cuidados Intensivos (UCI)? ____ Sí ____ No

***[If yes, ask the following questions. Otherwise only ask the questions to the intervention group]***

¿Con qué frecuencia usted escribió en el diario? ____ veces a la semana

¿Escribió con la misma frecuencia durante la estancia en la UCI? ____ veces a la semana

¿Escribió con la misma frecuencia después del alta? ____ veces a la semana

**IF THE PATIENT IS LIVING:** ¿Usted le permitió a su ser querido leer el diario de la Unidad de Cuidados Intensivos (UCI)?

____ Sí ____ No

Si la respuesta es **Sí:** ¿Hay algo que usted quisiera contarnos sobre su experiencia compartiendo el diario de la Unidad de Cuidados Intensivos (UCI) con su ser querido? ____________________________________________________________________________________________________________________________________________________________

Si la respuesta es **NO**: ¿Piensa compartir el diario de la Unidad de Cuidados Intensivos (UCI) con su ser querido?

____ Sí ____ No

¿Hay algo que le haya gustado del diario de la Unidad de Cuidados Intensivos (UCI) que le gustaría compartir con nosotros? __________________________________________________________________________________________________________________________________________________________________________

¿Tuvo alguna dificultad que le gustaría compartir con nosotros? __________________________________________________________________________________________________________________________________________________________________________

Si usted tuviera que dar un consejo a alguien para escribir un diario de la Unidad de Cuidados Intensivos (UCI), ¿qué le diría? __________________________________________________________________________________________________________________________________________________________________________

Si usted pudiera cambiar algo del diario de la Unidad de Cuidados Intensivos (UCI), ¿qué cambiaría? __________________________________________________________________________________________________________________________________________________________________________

¿Hay algo más que usted quisiera compartir sobre su experiencia escribiendo en el diario? __________________________________________________________________________________________________________________________________________________________________________

**Por favor, valore su acuerdo con las siguientes afirmaciones.**

**1. Mis creencias religiosas y/o espirituales me han ayudado a afrontar el evento cardíaco de mi ser querido.**

□ Muy en desacuerdo □ En desacuerdo □ Ni de acuerdo ni en desacuerdo

□ De acuerdo □ Muy de acuerdo □ Prefiero no decirlo

**2. El evento cardíaco de mi ser querido ha debilitado mis creencias religiosas y/o espirituales.**

□ Muy en desacuerdo □ En desacuerdo □ Ni de acuerdo ni en desacuerdo

□ De acuerdo □ Muy de acuerdo □ Prefiero no decirlo

**3. El evento cardíaco de mi ser querido ha reforzado mis creencias religiosas y/o espirituales.**

□ Muy en desacuerdo □ En desacuerdo □ Ni de acuerdo ni en desacuerdo

□ De acuerdo □ Muy de acuerdo □ Prefiero no decirlo

**CONTACTO EN EL FUTURO**

1. ¿Está dispuesto(a) a ser contactado(a) por nuestro Centro sobre estudios de investigación en el futuro? (S/N)

(Si Respondió Sí a #1): ¿Está dispuesto(a) a ser contactado(a) por nuestro Centro en el futuro para proporcionar comentarios sobre el diseño de estudios de investigación desde la perspectiva de un(a) participante? (S/N)
