# Supplementary figures and images for: Family-authored ICU diaries to reduce fear in patients experiencing a cardiac arrest (FAID fear): A pilot randomized controlled trial
Source: PLoS One. 2023 Jul 27;18(7):e0288436. doi: 10.1371/journal.pone.0288436 (PMC10373992; doi:10.1371/journal.pone.0288436)

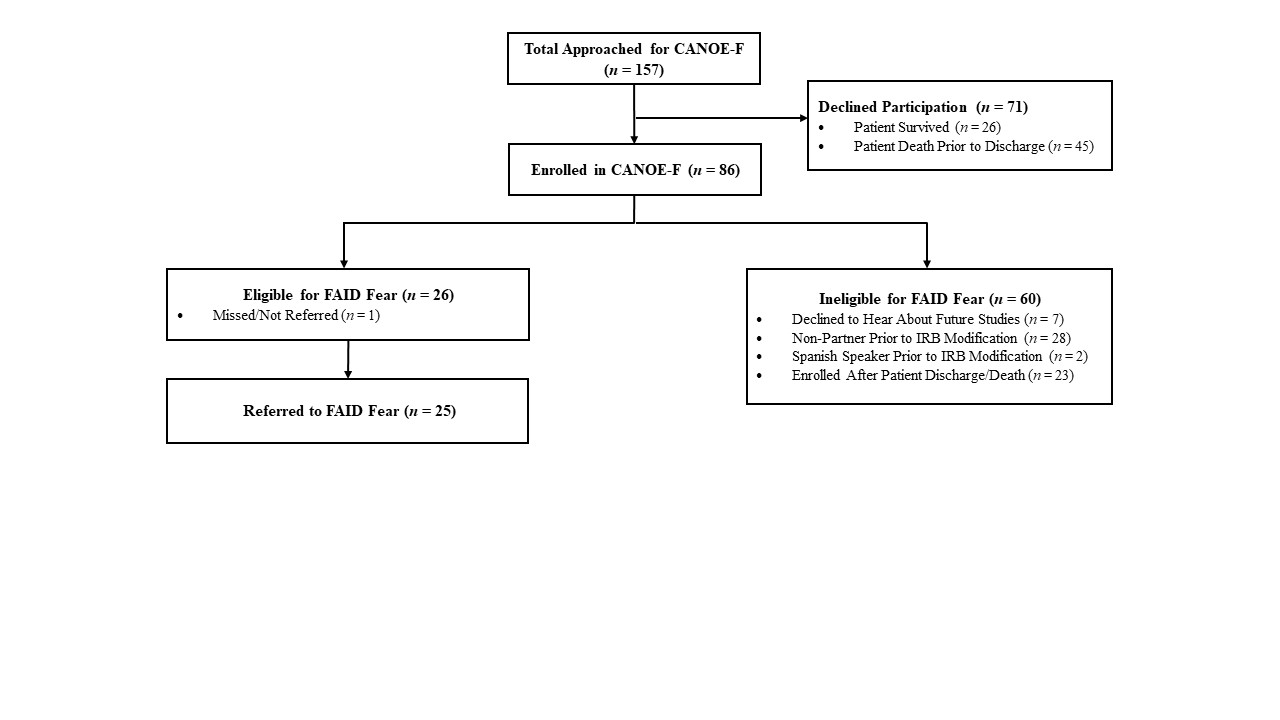

Supplement: S3 Appendix — (JPG) [file pone.0288436.s003.jpg]
